# A genetically small fetus impairs placental adaptations near term

**DOI:** 10.1242/dmm.050719

**Published:** 2024-08-29

**Authors:** Ionel Sandovici, Olatejumoye Knee, Jorge Lopez-Tello, Norman Shreeve, Abigail L. Fowden, Amanda N. Sferruzzi-Perri, Miguel Constância

**Affiliations:** ^1^ Department of Obstetrics and Gynaecology and National Institute for Health Research Cambridge Biomedical Research Centre, Cambridge CB2 0SW, UK; ^2^ Metabolic Research Laboratories and MRC Metabolic Diseases Unit, Wellcome Trust-MRC Institute of Metabolic Science, University of Cambridge, Cambridge CB2 0QQ, UK; ^3^ Centre for Trophoblast Research, Department of Physiology, Development and Neuroscience, University of Cambridge, Cambridge CB2 3EG, UK; ^4^ Department of Physiology, Faculty of Medicine, Universidad Autónoma de Madrid, Madrid 28029, Spain

**Keywords:** IGF2, Fetal growth restriction (FGR), Placenta, Placental nutrient transfer, Umbilical artery

## Abstract

The placenta is a gatekeeper between the mother and fetus, adapting its structure and functions to support optimal fetal growth. Studies exploring adaptations of placentae that support the development of genetically small fetuses are lacking. Here, using a mouse model of impaired fetal growth, achieved by deleting insulin-like growth factor 2 (*Igf2*) in the epiblast, we assessed placental nutrient transfer and umbilical artery (UA) blood flow during late gestation. At embryonic day (E) 15.5, we observed a decline in the trans-placental flux of glucose and system A amino acids (by using ^3^H-MeG and ^14^C-MeAIB), proportionate to the diminished fetal size, whereas UA blood flow was normal. However, at E18.5, the trans-placental flux of both tracers was disproportionately decreased and accompanied by blunted UA blood flow. Feto-placental growth and nutrient transfer were more impaired in female conceptuses. Thus, reducing the fetal genetic demand for growth impairs the adaptations in placental blood flow and nutrient transport that normally support the fast fetal growth during late gestation. These findings have important implications for our understanding of the pathophysiology of pregnancies afflicted by fetal growth restriction.

## Teaser

**Summary:** In a mouse model of reduced fetal growth demand, the placenta receives diminished blood flow via the umbilical artery and lowers its nutrient transfer capacity towards the end of gestation.

## INTRODUCTION

Appropriate fetal growth hinges upon cooperative interplay between the mother, fetus and placenta during the entire course of pregnancy (Constância et al., 2002, 2005; Coan et al., 2008a,b; Angiolini et al., 2011; Sferruzzi-Perri et al., 2016; López-Tello et al., 2019, 2023). The mechanisms underpinning this cooperation have a dual nature in ensuring a successful pregnancy outcome and in adapting to adverse environmental circumstances (Doherty et al., 2003; Sferruzzi-Perri et al., 2013; Higgins et al., 2016; Musial et al., 2017). As the interface between the mother and fetus, the placenta must be able to adapt to alterations in maternal physiology, as well as to dynamic changes in fetal growth rates (Sandovici et al., 2012; O'Brien and Wang, 2023). Placental adaptations are both structural (e.g. changes in placental vasculature) and functional (e.g. changes in trans-placental nutrient transfer) and designed to optimize fetal growth and enhance viability at birth in the prevailing environmental conditions, often with consequences for the health of the offspring in adulthood (Sandovici et al., 2012; Burton et al., 2016).

According to the International Federation of Gynecology and Obstetrics (FIGO), the term ‘small for gestational age’ (SGA) is employed to describe a constitutionally healthy fetus that is naturally smaller in size, with an estimated fetal weight falling below the 10th percentile for its gestational age. By contrast, fetal growth restriction (FGR) characterizes a small fetus that has not attained its full growth potential, typically due to an underlying pathological process (Melamed et al., 2021). To more accurately identify those fetuses that do not fulfil their growth potential and are at risk of poor outcomes, the 2015 Delphi consensus defined FGR as birth weight below the 3rd percentile (Gordijn et al., 2016). In a more recent update of the Delphi procedure, placental weight below the 10th percentile has been added as a contributory variable (Beune et al., 2021). FGR associates a wide range of morphological and functional placental alterations (Sun et al., 2020). However, although the pathophysiology of placental-derived FGR, primarily due to deficient remodeling of the uterine spiral arteries during early pregnancy, is clearer (Burton and Jauniaux, 2018), lack of appropriate models make it more difficult to assess placental adaptations in pregnancies with genetically small fetuses.

The feto-placental vasculature is key for successful placental functional adaptations during pregnancy, mediating the transfer of nutrients and oxygen required for normal fetal growth (Reynolds et al., 2010; Burton and Fowden, 2015; Sferruzzi-Perri et al., 2023). In human pregnancy, abnormalities in the feto-placental vasculature are frequently observed at term in compromised pregnancies (i.e. when fetal growth is altered), including in those resulting from maternal nutritional imbalances (Kadyrov et al., 1998; Gernand et al., 2013), environmental stress (Levine et al., 2017), advanced maternal age (Cooke and Davidge, 2019; Biagioni et al., 2021), multiparity (Ballering et al., 2018) or the use of assisted reproductive technologies (Delbaere et al., 2007; Zhang et al., 2011; Schroeder et al., 2022).

Animal models that use controlled genetic manipulations allow inferring mechanistic links between patterns of fetal growth and placental morphological, and functional adaptations. Similar to humans, the mouse has a hemochorial placental structure with a defined feto-maternal exchange barrier, referred to as the labyrinthine zone (Lz), which exhibits characteristics that resemble the human villous tree (Georgiades et al., 2002; Carter, 2007). Owning to the ease of genetic manipulations and similarities in the structure of the placental interchange barrier, the mouse has emerged as a good model for investigating the intricate interplay between the fetal and placental phenotypes. Although the number of genetic mutations in mice that elicit abnormal placental morphology has increased, only a few studies have used such models to study placental nutrient transfer in instances of altered feto-placental vasculature (Sekita et al., 2008; Wyrwoll et al., 2009; Stanley et al., 2012). Studies on feto-placental blood flow in models with altered placental vasculature are also scarce (Singh et al., 2011; Kulandavelu et al., 2013; Rennie et al., 2015; Yamaleyeva et al., 2015; Cahill et al., 2019). Moreover, none of these studies assessed placental nutrient transfer and feto-placental blood flow in combination, and very few have made repeated measurements across gestation, which is critical for understanding pathogenesis of FGR.

Insulin-like growth factor 2 (IGF2), encoded by the paternally expressed imprinted *Igf2* gene, is a major factor controlling pre-natal growth (Sferruzzi-Perri et al., 2017; Sélénou et al., 2022). In humans, levels of IGF2 in the umbilical cord blood show a weak positive association with both fetal and placental weights (Luo et al., 2012). Additionally, SGA babies have been reported to show reduced levels of IGF2 in the umbilical cord blood (Tzschoppe et al., 2015). We have previously generated a mouse model with reduced fetal demand for growth, achieved by deleting *Igf2* in the epiblast (*Igf2*^EpiKO^) (Sandovici et al., 2022). The epiblast gives rise to the entire fetus, as well as to the feto-placental capillaries (Tallquist and Soriano, 2000). In this conditional knockout model, FGR occurs as early as embryonic day (E) 11.5, with placental growth restriction first detectable at E13.5. The small *Igf2*^EpiKO^ placenta is characterized by a diminished Lz expansion in the late stages of pregnancy, with a disproportional reduction in feto-placental capillaries (Sandovici et al., 2022). Based on these observations, we hypothesized that the *Igf2*^EpiKO^ placenta, supporting a fetus with diminished genetic demand for growth, has a reduced capacity to transfer nutrients from the mother to the fetus. To test this hypothesis and uncover the mechanisms involved, we characterized the morphological, nutrient-transfer and blood-flow properties of *Igf2*^EpiKO^ placentae during late gestation. Specifically, we used stereological techniques to estimate theoretical placental diffusion capacity, placental transfer assays to determine facilitated diffusion and active transport of nutrients and ultrasound imaging to provide an *in vivo* estimation of umbilical artery (UA) blood flow.

## RESULTS

### The genetically small *Igf2*^EpiKO^ fetus impairs normal placental growth during late gestation, with stronger impact on females

**Fig. 1.**
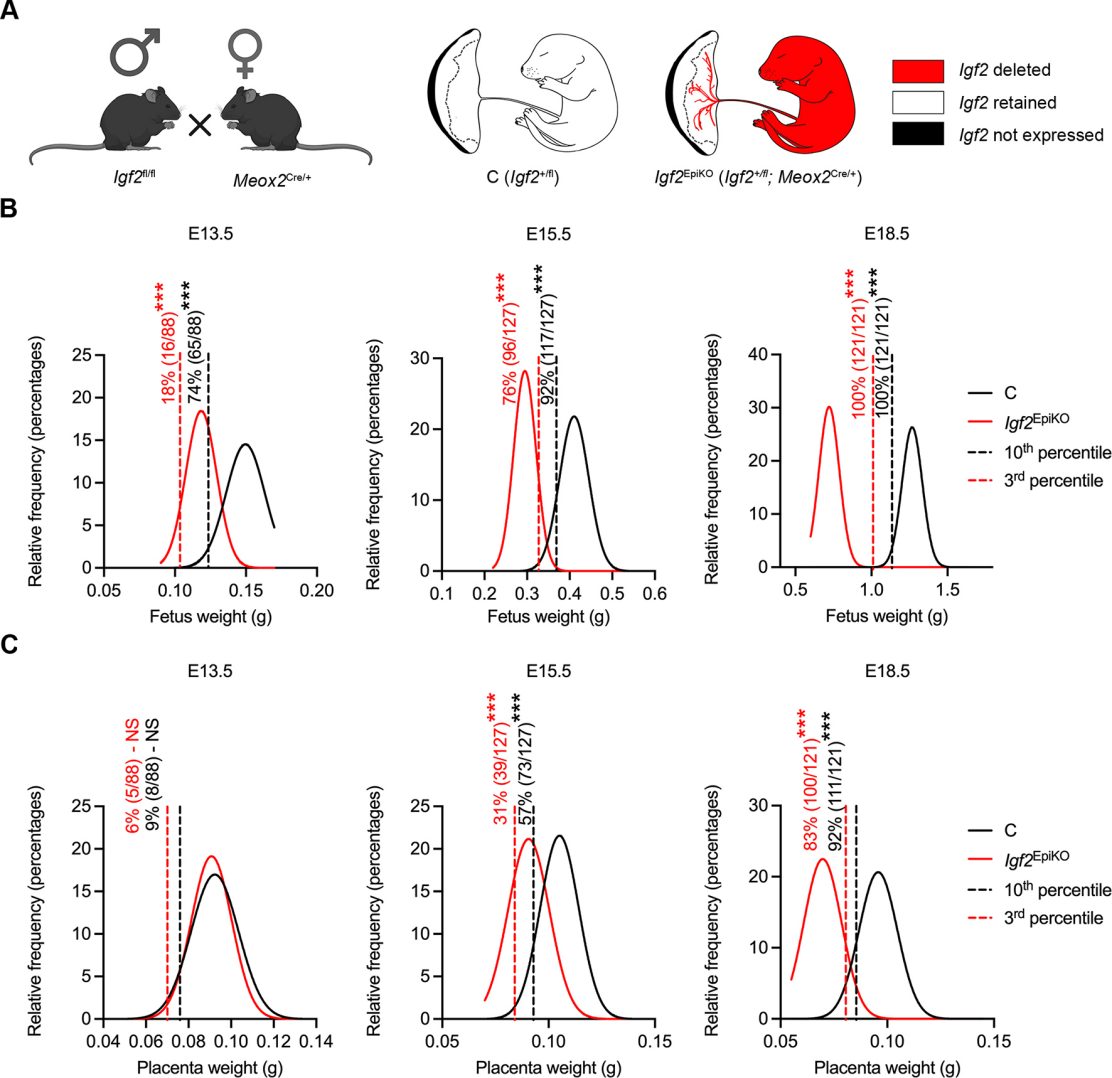
**Fetal and placental weight distribution curves for *Igf2*^EpiKO^ mutants versus littermate controls.** (A) Diagram depicting the mating strategy used to achieve conditional deletion of the paternally expressed *Igf2* allele in the epiblast (*Igf2*^EpiKO^). (B) Fetal weight distribution curves for *Igf2*^EpiKO^ mutants versus littermate controls, showing an excess of *Igf2*^EpiKO^ fetuses below the 10th and the 3rd percentiles. (C) Placenta weight distribution curves for *Igf2*^EpiKO^ mutants versus littermate controls, showing an excess of *Igf2*^EpiKO^ placentae below the 10th and the 3rd percentiles. For panels B and C, to apply a best-fit Gaussian line, placental and fetal weights were first organized in bins, according to their frequency distribution, with least-squares fit. The distribution curves in panels B and C were generated from *N*=25 litters at E13.5 (*n*=114 C and *n*=88 *Igf2*^EpiKO^), *N*=37 litters at E15.5 (*n*=154 C and *n*=127 *Igf2*^EpiKO^) and *N*=37 litters at E18.5 (*n*=164 C and *n*=121 *Igf2*^EpiKO^). For both panels, the black and the red dashed lines represent the 10th and the 3rd percentile of the control curves, respectively. Values in percent indicate the fractions of fetuses and placentae that fall below these thresholds (absolute numbers are provided in parentheses). ***P<0.001 (Fisher's exact test).

To further characterize the previously reported fetal and placental growth restriction of mutants that lack *Igf2* expression in epiblast-derived structures (*Igf2*^EpiKO^, Fig. 1A), we generated fetal and placental weight distribution curves across the late stages of pregnancy. We then calculated the percentages of mutant fetuses and placentae that were below the 10th and the 3rd percentiles at each of the three time points assessed in this study, i.e. E13.5, E15.5 and E18.5 (Fig. 1B,C). At E13.5, there was a significant excess of both small (<10th percentile) and growth-restricted (<3rd percentile) *Igf2*^EpiKO^ fetuses (Fig. 1B), whereas placenta weight distribution was similar between control (C) and *Igf2*^EpiKO^ conceptuses (Fig. 1C).

At E15.5, there were significant excesses of both *Igf2*^EpiKO^ fetuses and placentae below the 10th and 3rd percentiles; however, these percentages were higher for fetuses than for placentae (Fig. 1B,C). At E18.5, all *Igf2*^EpiKO^ fetuses were below the 3rd percentile, whereas – proportionately – more of the placentae were below the 10th and 3rd percentiles than at E15.5 (Fig. 1B,C). When considering the sex of the conceptus, data analysis uncovered a higher proportion of females among growth-restricted *Igf2*^EpiKO^ fetuses at both E13.5 and E15.5 (Fig. S1). In addition, at E15.5, the proportion of placentae below the 10th and 3rd percentiles conceptuses was increased among *Igf2*^EpiKO^ female conceptuses compared to male conceptuses (Fig. S2). These data are consistent with our previous findings that fetus-derived IGF2 controls placenta growth during late gestation (Sandovici et al., 2022) and demonstrate a stronger effect in female conceptuses.

### *Igf2*^EpiKO^ placentae are associated with a reduction in the theoretical diffusion capacity

**Fig. 2.**
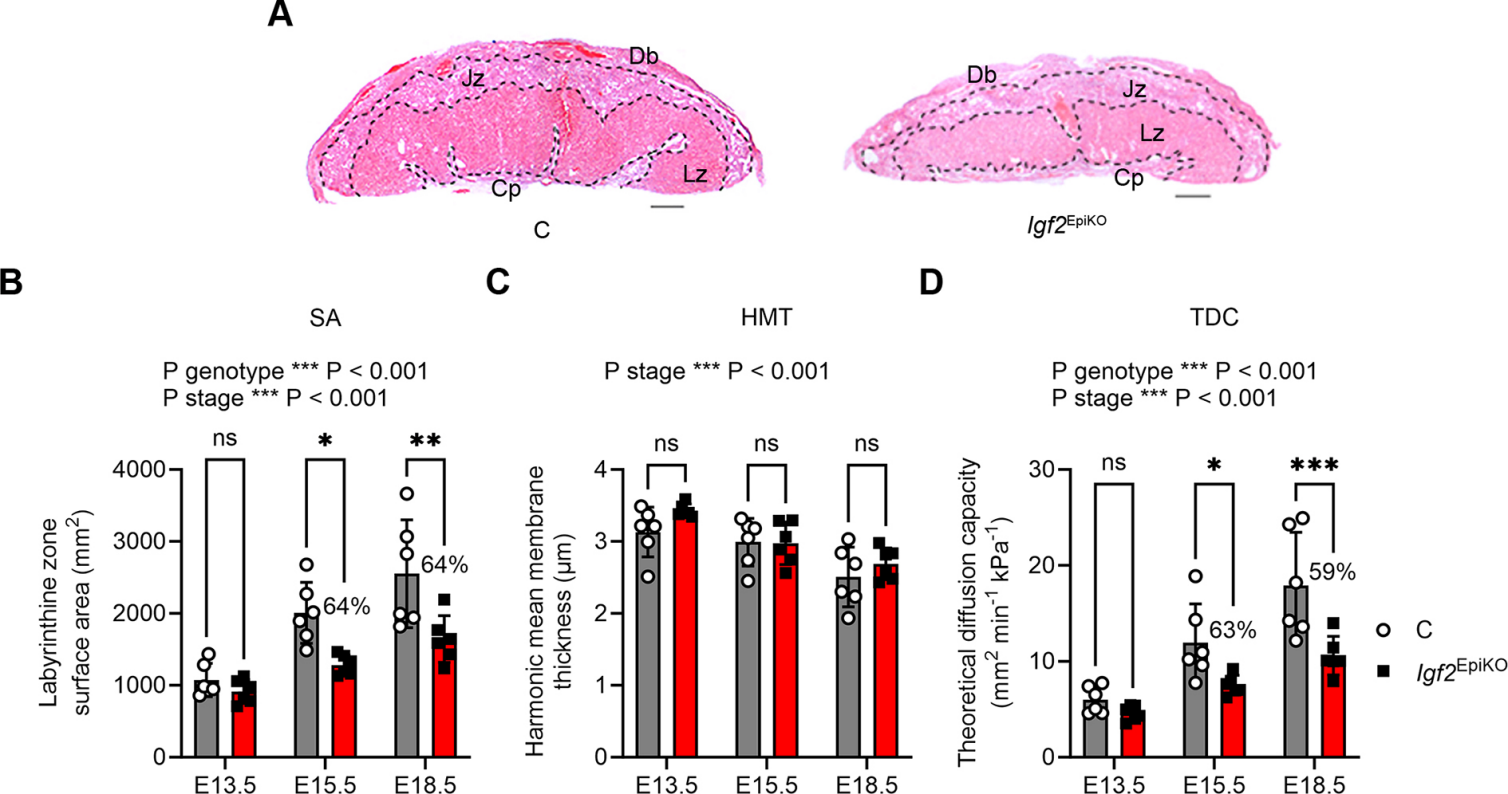
**Stereological analysis of the placental labyrinthine zone in *Igf2*^EpiKO^ mutants versus littermate controls.** (A) Representative hematoxylin-eosin staining of placental mid-sections obtained from E18.5 controls (C; left) and *Igf2*^EpiKO^ mutants (right), illustrating the disproportional growth restriction of the nutrient exchange layer. Lz, labyrinthine zone; Db, decidua basalis; Jz, junctional zone; Cp, chorionic plate. Scale bars: 500 µm. (B-D) Stereological measurements of (B) labyrinthine zone surface area (SA), (C) harmonic mean membrane thickness (HMT) and (D) theoretical diffusion capacity (TDC), showing reduced area available for nutrient transfer in *Igf2*^EpiKO^ placentae at the end of gestation. In all graphs, data are shown as individual data points (*n*=6 biological samples per group at each developmental stage). Error bars indicate the mean±standard deviation (±s.d). *P*-values shown above each graph indicate statistically significant two-way ANOVA tests. Statistical differences between the two genotypes at each developmental stage were calculated using Sidak's multiple comparison tests following two-way ANOVA (*P<0.05, **P<0.01, ***P<0.001; ns, non-significant). Values in percent indicate the *Igf2*^EpiKO^/C ratios.

By using stereological measurements, our previous work has demonstrated that, from E15.5 onwards, the smaller Lz of *Igf2*^EpiKO^ placenta (Fig. 2A) has a reduced volume regarding all its structural components – i.e. fetal capillaries, maternal blood spaces and labyrinthine trophoblasts (Sandovici et al., 2022). To evaluate the nutrient transfer capacity of the placenta, we first calculated the interhemal surface area, as a mean of fetal capillary and maternal blood space surfaces (see Materials and Methods). Whereas the control group exhibited the anticipated linear increase in the interhemal surface area with advancing gestational age, this expansion was notably blunted in *Igf2*^EpiKO^ placentae, with significant reductions by approximately one-third in the interhemal surface area at both E15.5 and E18.5 (Fig. 2B).

Another parameter that is critical for placental capacity to transfer nutrients and gases is the thickness of the interhemal membrane. The harmonic mean barrier thickness (HMT) showed the well-established thinning during late gestation (Coan et al., 2004), without any significant differences between placentae of *Igf2*^EpiKO^ mutants and littermate controls (Fig. 2C). Last, we calculated the theoretical diffusion capacity (TDC) of the Lz interhemal membrane. As expected (Coan et al., 2004), there was a significant increase of TDC from E13.5 to E18.5 in control placentae. However, the TDC increase over this period was blunted in *Igf2*^EpiKO^ placentae, with significant reductions in TDC at both E15.5 and E18.5 relative to their controls (Fig. 2D). Taken together, these observations strongly suggest that the small Lz of *Igf2*^EpiKO^ placentae have a reduced capacity to transfer nutrients and oxygen during late gestation.

### *Igf2*^EpiKO^ placentae transport glucose proportional to the fetal demand

**Fig. 3.**
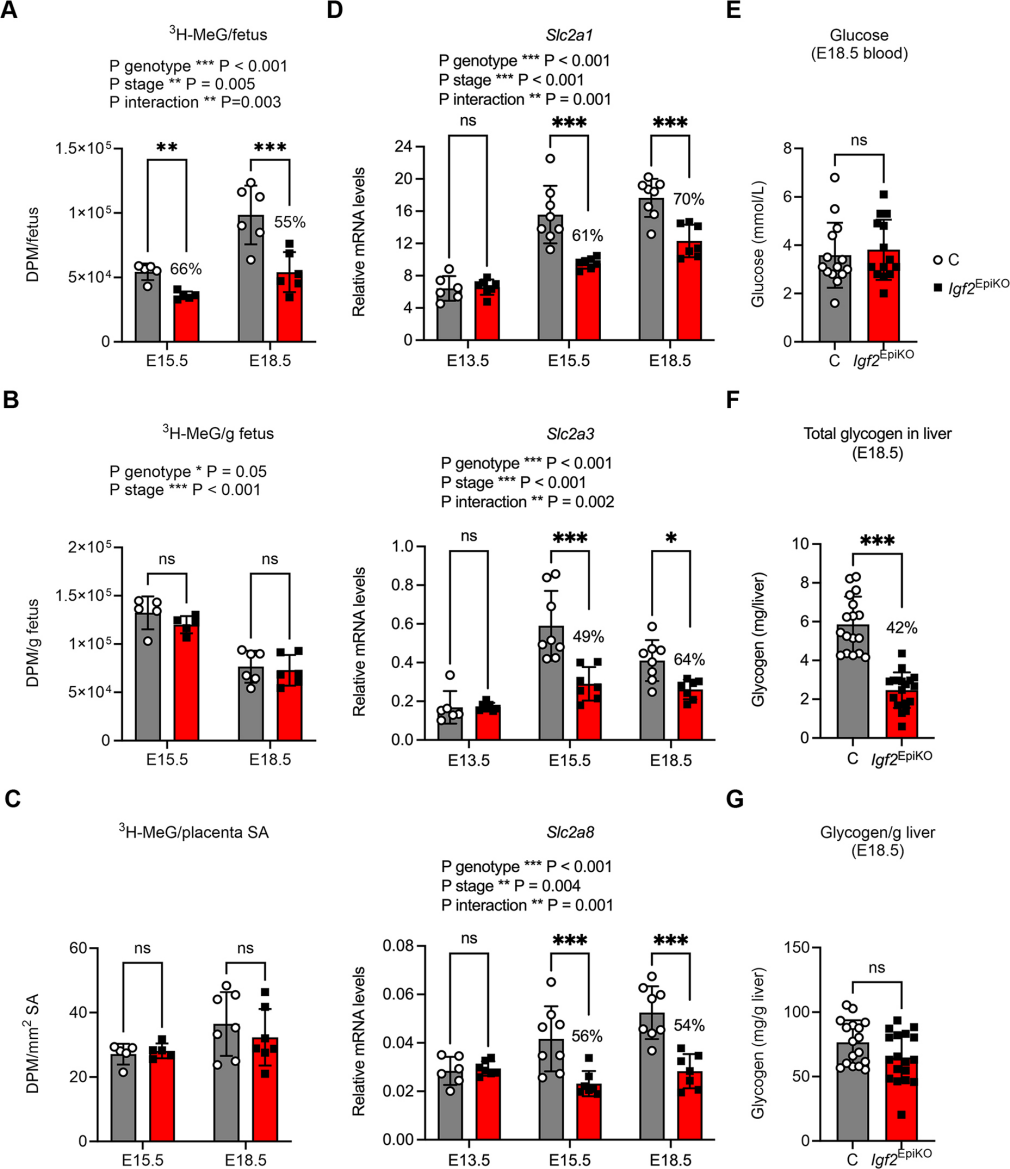
**Placental transfer of ^3^H-methyl-D-glucose (^3^H-MeG), as marker of facilitated transport, and its impact on fetal glucose metabolism.** (A-C) Measurements of disintegrations per minute (DPM) in the fetus, represented as beta-particle counts per (A) fetus, (B) gram fetal weight and (C) mm^2^ labyrinthine zone surface area (SA). Placental transfer of ^3^H-MeG in *Igf2*^EpiKO^ mutants is largely proportionate with the reduced Lz SA. Data in panels A-C were collected in *N*=5 litters at E15.5 (*n*=22 C and *n*=22 *Igf2*^EpiKO^ mutants) and *N*=7 litters at E18.5 (*n*=30 C and *n*=29 *Igf2*^EpiKO^ mutants). (D) Quantification of relative mRNA levels of *Slc2a1* (top) *Slc2a3* (middle) and *Slc2a8* (bottom) measured in micro-dissected female placental labyrinthine zone samples, relative to three housekeeping genes (*Gapdh*, *Pmm1*, *Sdha*). All three genes have reduced expression levels at E15.5 and E18.5 in *Igf2*^EpiKO^ Lz placentae. E13.5: *n*=6 (control), *n*=7 (*Igf2*^EpiKO^ mutant); E15.5: *n*=8 (control), *n*=7 (*Igf2*^EpiKO^ mutant); E18.5: *n*=8 Quantification of *n*=7 (*Igf2*^EpiKO^ mutant)]. (E) Quantification of glucose levels (in mmol/L) measured in fetal blood obtained from samples collected from *N*=14 litters at E18.5 [*n*=14 (control, C), *n*=14 *Igf2*^EpiKO^ mutant)]. (F) Quantification of glycogen deposits in fetal livers of control (C) and *Igf2*^EpiKO^ mutant litters at E18.5, represented as total amount (in mg) per liver. (G) Quantification of glycogen deposits in fetal livers of control (C) and *Igf2*^EpiKO^ mutant litters at E18.5, normalized per unit (in mg/g) of liver weight. Data in panels F and G were obtained from samples collected from *N*=4 litters at E18.5 [*n*=17 (control, C), *n*=18 (*Igf2*^EpiKO^ mutant)]. For all graphs, data are presented as average values per litter (A-C) or individual data points (D-J). Error bars indicate the mean ±s.d. *P*-values shown above graphs correspond to statistically significant repeated measures two-way ANOVA (A-C) or two-way ANOVA (D). Statistical differences between the two genotypes at each developmental stage were calculated using Sidak's multiple comparison test following two-way ANOVA (A-D), Mann–Whitney test (E) or two-tailed unpaired Student's *t*-tests with Welch's correction (F,G) (*P<0.05, **P<0.01, ***P<0.001; ns, non-significant). Values in percent indicate the *Igf2*^EpiKO^/C ratios.

To directly evaluate the exchange properties of *Igf2*^EpiKO^ placentae we performed placental transfer assays (Fig. S3A) at E15.5 and E18.5, i.e. when growth is at its fastest in terms of absolute weight gain and *Igf2*^EpiKO^ placentae associate reduced TDC (Fig. 2D). First, we measured facilitated diffusion by quantifying the *in vivo* flux of the tracer ^3^H-methyl-D-glucose (^3^H-MeG), a non-metabolizable glucose analogue (Constância et al., 2005). Initially, we performed these analyses by averaging measurements recorded within a litter per genotype, irrespective of the fetal sex. Thus, accumulation of ^3^H-MeG per fetus was significantly reduced in *Igf2*^EpiKO^ mutants at both E15.5 and E18.5 (Fig. 3A), in line with the previously observed FGR at these gestational ages (Sandovici et al., 2022). Additionally, there was a small but significant reduction in the accumulation of ^3^H-MeG per gram of fetus across the two gestational time points (P genotype=0.05) (Fig. 3B). The transfer of ^3^H-MeG measured per mm^2^ Lz surface area was similar in the two genotypes at both gestational ages (Fig. 3C). However, analysis of data according to the fetal sex revealed a more severe impact on the trans-placental transfer of glucose in female compared to male *Igf2*^EpiKO^ mutants, including a significant reduction of ^3^H-MeG measured per mm^2^ Lz surface area (Fig. S4). Measurements performed in placenta irrespective of fetal sex demonstrated that, although accumulation of ^3^H-MeG per placenta was significantly less in *Igf2*^EpiKO^ mutants at E18.5 (Fig. S3B), *Igf2*^EpiKO^ placentae accumulated an appropriate amount of tracer for their weight at both E15.5 and E18.5 (Fig. S3C).

We then measured in female placental Lz samples the mRNA levels of three transporters that mediate the trans-placental transfer of glucose: *Slc2a1* (also known as GLUT1) and *Slc2a3* (also known as GLUT3) that are highly expressed in mouse placenta (Constância et al., 2005) and *Slc2a8* (also known as GLUT8), previously identified as downregulated at E18.5 in placental Lz samples of male *Igf2*^EpiKO^ mutants by expression microarray analysis (Sandovici et al., 2022). At E13.5, all three genes had similar mRNA levels in the two genotypes (Fig. 3D). However, at E15.5 and E18.5, the *Igf2*^EpiKO^ placentae had significantly lower mRNA levels for all three genes compared to control littermate controls (Fig. 3D).

Subsequently, we assessed the impact of glucose transfer by facilitated diffusion on fetal and placental glucose homeostasis. Fetal blood glucose concentrations were similar between the two genotypes at E18.5 (Fig. 3E). Although total glycogen content of the fetal liver was less in *Igf2*^EpiKO^ mutants at E18.5 (Fig. 3F), this level was appropriate for the disproportionally reduced liver weight (Fig. 3G; Fig. S5). Similarly, total placental glycogen reserve was less in *Igf2*^EpiKO^ mutants at E18.5 (Fig. S3D) but, again, this reduction aligned with the lower placental weight of the mutants (Fig. S3E,F).

Based on these measurements, we conclude that the trans-placental flux of glucose through facilitated diffusion is reduced in *Igf2*^EpiKO^ mutants during late gestation – to levels that are largely proportionate to the diminished fetal demand – and provide evidence for sex-specific effects.

### *Igf2*^EpiKO^ placentae have disproportionally reduced amino acid transport at the end of gestation

**Fig. 4.**
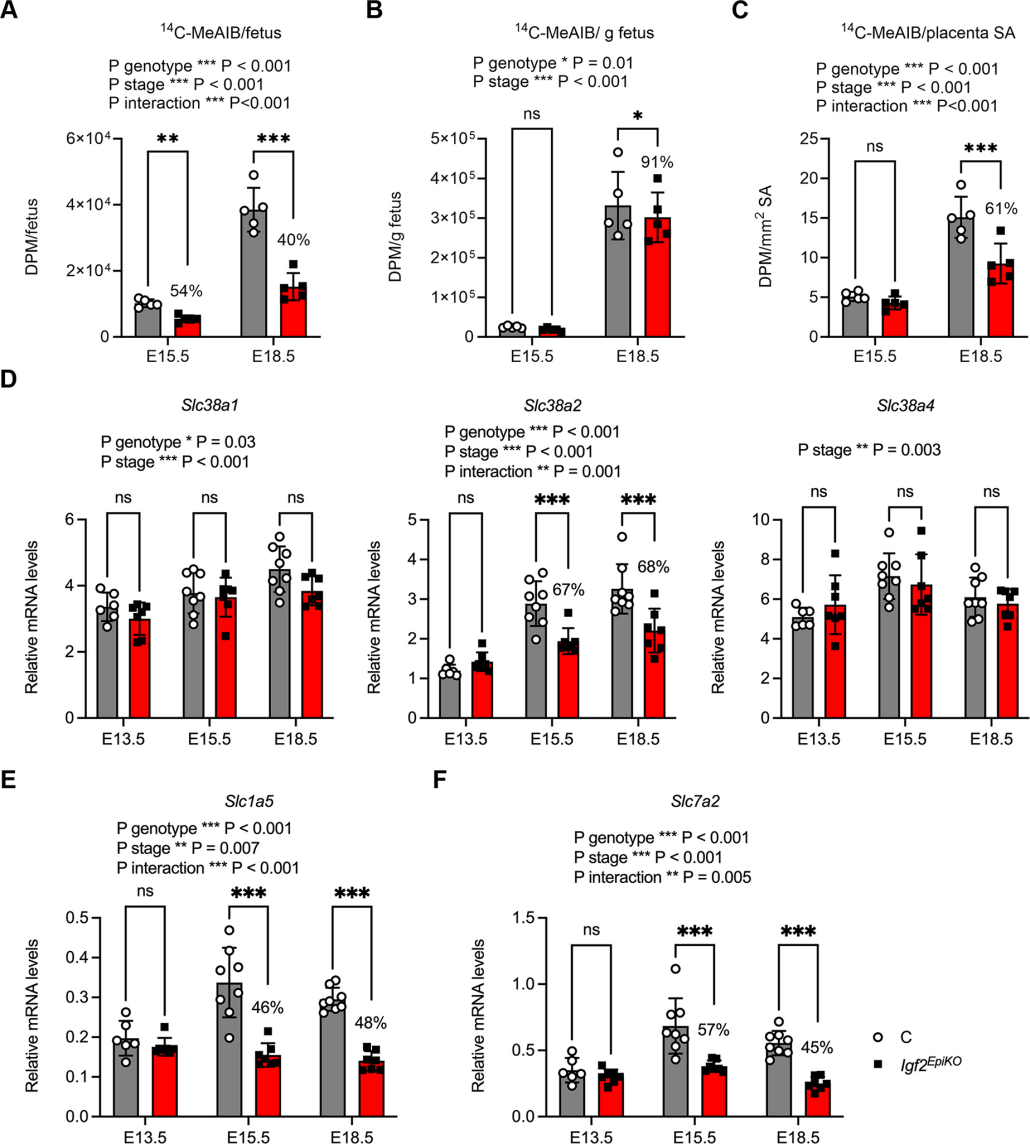
**Placental transfer of ^14^C-methyl-aminoisobutyric acid (^14^C-MeAIB), as marker of active transport mediated by system A amino acid placental transporters.** (A-C) Measurement of disintegrations per minute (DPM) in the fetus, represented as beta-particle counts per (A) fetus, (B) gram fetal weight and (C) mm^2^ labyrinthine zone surface area (SA). Data in panels A-C were collected in *N*=5 litters at E15.5 (*n*=22 C and *n*=22 *Igf2*^EpiKO^ mutants) and *N*=5 litters at E18.5 (*n*=22 C and *n*=15 *Igf2*^EpiKO^ mutants). Placental transfer of ^14^C-MeAIB in *Igf2*^EpiKO^ mutants is disproportionately reduced at E18.5, even after taking into account the reduced fetal weight and the smaller placenta SA. (D-F) Quantification of relative mRNA levels of *Slc38a1*, *Slc38a2* and *Slc38a4* (D), *Slc1a5* (E) and *Slc7a2* (F) measured in micro-dissected female placental labyrinthine zone samples, relative to three housekeeping genes (*Gapdh*, *Pmm1*, *Sdha*). These data show reduced expression in placental Jz of *Igf2*^EpiKO^ mutants at the end of gestation of specific genes encoding amino acid transporters. Data in panels D-E were obtained from samples collected from *N*=3 litters [E13.5: *n*=6 (control), *n*=7 (*Igf2*^EpiKO^ mutant); E15.5: *n*=8 (control), *n*=7 (*Igf2*^EpiKO^ mutant); E18.5: *n*=8 (control), *n*=7 (*Igf2*^EpiKO^ mutant)] and are shown as average per litter (A-C) or individual values (D-F). Error bars indicate the mean ±s.d. *P*-values shown above graphs correspond to statistically significant repeated measures two-way ANOVA (A-C) or two-way ANOVA (D-F). Statistical differences between the two genotypes at each developmental stage were calculated using Sidak's multiple comparison tests following repeated measures two-way ANOVA (A-C) or two-way ANOVA (D-F). (*P<0.05, **P<0.01, ***P<0.001; ns, non-significant). Values in percent indicate the *Igf2*^EpiKO^/C ratios.

Next, we investigated if active trans-placental transport of amino acids is altered in *Igf2*^EpiKO^ mutants. We measured the *in vivo* flux of the radioactive tracer ^14^C-methylaminoisobutyric acid (^14^C-MeAIB), a non-metabolizable amino acid analogue that is a specific substrate for system A amino acid transporters, a sodium-dependent transport system for neutral amino acids (Constância et al., 2005). Measurements recorded within a litter, irrespective of the fetal sex, showed that accumulation of ^14^C-MeAIB per fetus is significantly and severely reduced in *Igf2*^EpiKO^ mutants at both E15.5 and E18.5 (Fig. 4A). When normalized per gram of fetus (Fig. 4B) or per mm^2^ Lz surface area (Fig. 4C), levels of ^14^C-MeAIB measured in *Igf2*^EpiKO^ fetuses were significantly reduced only at E18.5. Similar to the observations made for the trans-placental flux of ^3^H-MeG, analysis of data according to the fetal sex revealed a more severe impact on transport of ^14^C-MeAIB in female *Igf2*^EpiKO^ mutants (Fig. S6). Total levels of ^14^C-MeAIB measured in *Igf2*^EpiKO^ placentae irrespective of fetal sex were significantly reduced at both E15.5 and E18.5 (Fig. S7A), and remained disproportionally low at E18.5 after accounting for the reduced placental weights (Fig. S7B).

System A amino acid transporters comprises three genes (*Slc38a1* encoding transporter SNAT1, *Slc38a2* encoding SNAT2 and *Slc38a4* encoding SNAT4) and is a major transporter system of amino acids in all cells. All three genes are highly expressed in placenta, where they play important roles in the supply of amino acids to the fetus (Constância et al., 2005). To identify which of the three system A members might mediate the reduced trans-placental transfer of ^14^C-MeAIB observed in *Igf2*^EpiKO^ mutants, we measured their expression at mRNA level in female placental Lz samples. We performed these measurements in placental Lz isolated at three gestational stages, i.e. E13.5, E15.5 and E18.5. At E13.5, all three genes had similar levels of mRNA in the two genotypes (Fig. 4D). However, at E15.5 and E18.5, *Igf2*^EpiKO^ placentae had significantly reduced mRNA levels for *Slc38a2* and a tendency of low mRNA levels for *Slc38a1*, whereas levels of *Slc38a4* mRNA remained comparable to that of control littermates (Fig. 4D).

The impact on amino acid transport in *Igf2*^EpiKO^ mutants is likely to extend beyond the system A amino acid transporters. We have previously identified two other amino acid transporter genes that were downregulated at E18.5 in placental Lz samples of male *Igf2*^EpiKO^ mutants, namely *Slc1a5* (encoding the neutral amino acid transporter ASCT2, part of amino acid transport system ASC) and *Slc7a2* (encoding the cationic amino acid transporter CTR2, part of amino acid transport system L) (Sandovici et al., 2022). In *Igf2*^EpiKO^ mutants, measurement of these two genes in female placental Lz samples demonstrated normal mRNA levels at E13.5 but significant reductions at both E15.5 and E18.5 (Fig. 4E,F).

Taken together, these observations demonstrate a reduction in the active transport of system A amino acids by the *Igf2*^EpiKO^ placentae at E18.5, which is disproportionately lower than the concurrent fetal demand assessed as fetal weight and more severely affected in female conceptuses. This altered transport may be mediated by a reduced abundance of SNAT1 and SNAT2 transporters in placental Lz. Furthermore, our data also suggest a reduction in the amino acid transport via other systems, such as ASC and L.

### *Igf2*^EpiKO^ mutants have altered UA blood flow at the end of gestation

**Fig. 5.**
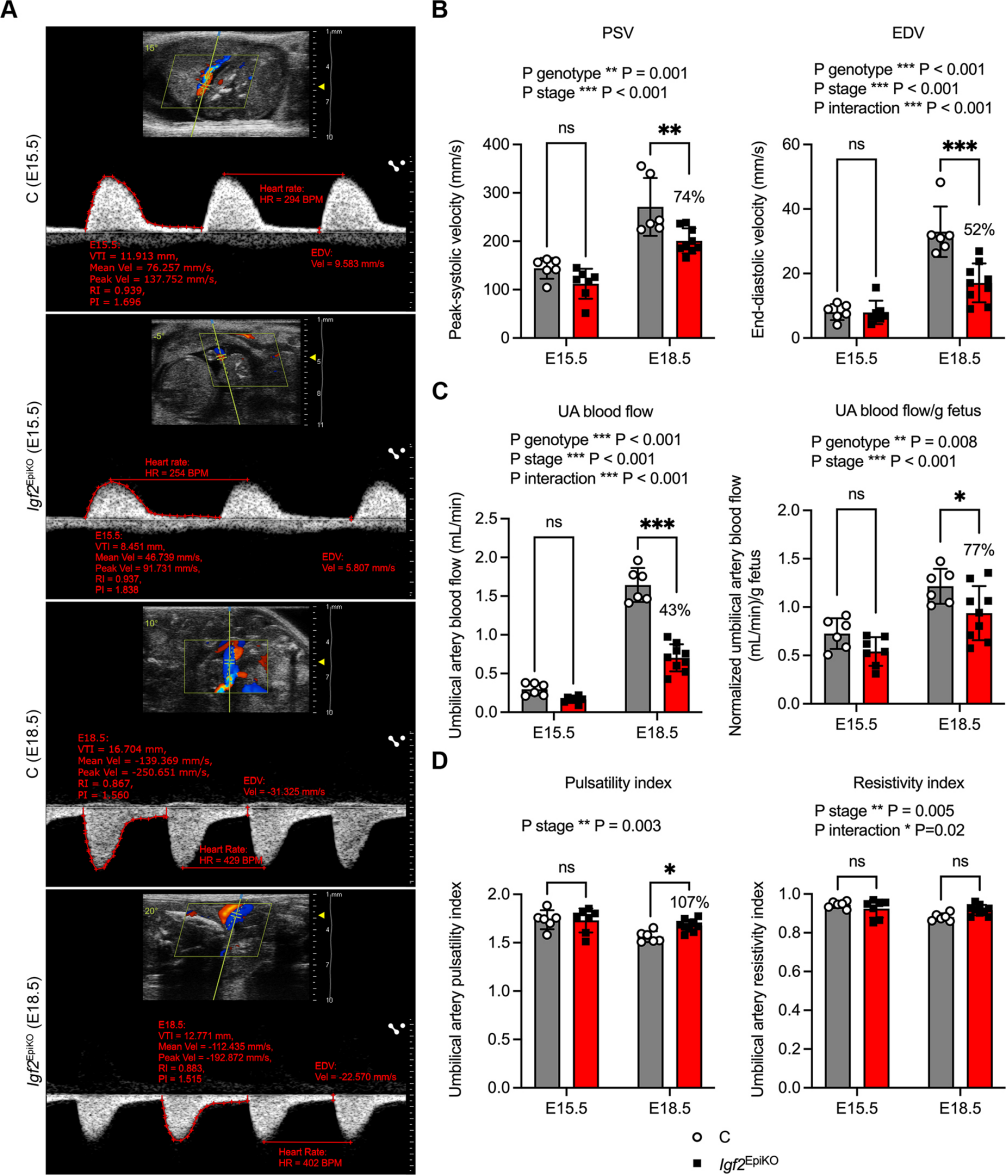
**Pulsed-wave Doppler ultrasonography assessment of the umbilical artery in *Igf2*^EpiKO^ mutants versus littermate controls.** (A) Representative Power Doppler images depicting the umbilical artery of E15.5 and E18.5 fetuses, used for offline data analysis. Notice the angle of <30° (shown next to the green parallelograms) between the ultrasound beam (solid green line) and direction of flow (dotted line). Blood flow direction is indicated by red (flow towards the transducer) and blue (flow away from the transducer). (B) Measurement of umbilical artery peak-systolic velocity (PSV) and end-diastolic velocity (EDV). Data show reduced UA blood velocities in *Igf2*^EpiKO^ mutants at E18.5. (C) Estimated UA blood flow and UA blood flow per gram fetus at E15.5 and E18.5. Data show reduced UA blood flow in *Igf2*^EpiKO^ mutants at E18.5. (D) Calculated pulsatility and resistivity indexes at E15.5 and E18.5. Data show increased UA PI in *Igf2*^EpiKO^ mutants at E18.5. For all graphs, data were obtained in *N*=3 litters at E15.5 (*n*=6 C and *n*=7 *Igf2*^EpiKO^ mutants) and *N*=3 litters at E18.5 (*n*=6 C and *n*=9 *Igf2*^EpiKO^ mutants); data are shown as individual data points with mean values±s.d. *P*-values shown above each graph indicate statistically significant two-way ANOVA tests. Statistical differences between the two genotypes at each developmental stage were calculated using Sidak's multiple comparison tests following two-way ANOVA (*P<0.05, **P<0.01, ***P<0.001; ns, non-significant). Values in percent indicate the *Igf2*^EpiKO^/C ratios.

To assess the potential contribution of placental blood flow to the reduced fetal growth and reduced placental nutrient transfer observed in *Igf2*^EpiKO^ mutants at the end of gestation, we conducted *in vivo* assessment of UA blood flow at E15.5 and E18.5 using high-frequency ultrasonography. In M-Mode (Fig. S8A), we noted that the inner diameter of the UA was diminished in *Igf2*^EpiKO^ mutants compared to that of control littermates at both E15.5 and E18.5, whereas outer UA diameter and wall thickness were significantly reduced only at E18.5 (Fig. S8B). Subsequently, using the pulsed-wave (PW) Doppler mode (Fig. 5A), we assessed UA blood velocities, i.e. PSV (peak-systolic velocity), EDV (end-diastolic velocity) and UA mean velocity. Although at E15.5 these three parameters were at similar levels in the two genotypes, at E18.5 they were significantly reduced in *Igf2*^EpiKO^ mutants compared with littermate controls (Fig. 5B; Fig. S9A). Additionally, we measured the velocity-time integral (VTI) (Fig. S9B) and the area of the UA lumen (Fig. S9C), both of which were reduced at E18.5 in *Igf2*^EpiKO^ mutants. By contrast, the fetal heart rate increased between E15.5 and E18.5 but remained similar between the two genotypes (Fig. S9D). Based on VTI, UA area and fetal heart rate measurements, we then estimated the UA blood flow (see Materials and Methods) and found a very profound (>50%) reduction in *Igf2*^EpiKO^ mutants at E18.5 (Fig. 5C). The UA blood flow remained reduced even after adjusting for the lower fetal weight (Fig. 5C). Pulsatility and resistivity indexes (PI and RI, respectively) were found to be lower at E18.5 than E15.5 (Fig. 5D). Nevertheless, despite the similarity of PI and RI for both genotypes at E15.5, *Igf2*^EpiKO^ mutants had a significantly higher PI at E18.5 (Fig. 5D). The RI, however, showed no significant differences between the two genotypes at E18.5 (Fig. 5D). Based on these findings, our conclusion is that *Igf2*^EpiKO^ mutants have umbilical arteries of reduced diameter during late gestation and display alterations in feto-placental blood flow, specifically at E18.5.

## DISCUSSION

The fetus has evolved intricate mechanisms to influence the development and functioning of the placenta, ensuring it aligns with its own demands for growth. In a recent study, we have identified fetus-derived IGF2 as a major signal facilitating communication of the fetal demands for growth to its own placenta (Sandovici et al., 2022). We have demonstrated that fetus-derived IGF2 acts as an endocrine factor and plays a pivotal role in orchestrating feto-placental capillary branching during late gestation, effects mediated at least in part via IGF2R–ERK1/2–angiopoietin−TEK signaling. In addition, fetus-derived IGF2 influences the growth and differentiation of the underlying trophoblast layer by regulating the expression levels of the key trophoblast differentiation genes *Gcm1* and *Synb* (Sandovici et al., 2022). In our current study, we set out to characterize the adaptations of the *Igf2*^EpiKO^ placenta supplying nutrients to a fetus with a genetically determined reduced demand for growth. Our observations allowed us to define several important mechanistic principles that govern placental adaptations in this mouse mutant.

First, we found that placental adaptations to the reduced fetal demand for growth in the *Igf2*^EpiKO^ model manifest as morphological changes, particularly evident at E15.5 by the reduction of Lz surface area and UA inner diameter. Interestingly, despite the reduction of Lz surface area during late gestation, the thickness of the interhemal membrane was maintained within physiological parameters throughout the late gestation in this model. In prior studies, we have reported an increase in the harmonic mean barrier thickness within two other *Igf2* genetic models, namely *Igf2*P0^+/−^ and *Igf2*null^+/−^ (Sibley et al., 2004; Coan et al., 2008a). Different to the previous two models, the *Igf2*^EpiKO^ placenta lacks *Igf2* only in the feto-placental endothelial cells (Sandovici et al., 2022). The findings of this current study further underscore the pivotal role of the trophoblast-derived *Igf2*P0 transcript – which was not deleted in this model – in regulating the diffusional exchange characteristics of the mouse placenta (Sibley et al., 2004). Furthermore, the reduced inner diameter of the UA, measured by using ultrasound imaging in our study, is consistent with earlier findings in the *Igf2*null^+/−^ model, which has been reported to have a reduced radius of the UA lumen (Ahmad et al., 2005).

Second, we observed evidence for functional placental adaptations that come into play at the end of pregnancy. Glucose and amino acids serve as the principal nutrients utilized by the fetus to drive energy production and support growth (O'Brien and Wang, 2023). At E15.5, the trans-placental transfer of both ^3^H-MeG and ^14^C-MeAIB appeared to be commensurate with the reduced size of the fetus for both sexes, and these transfers were proportional to the diminished Lz surface area. At E18.5, ^3^H-MeG and ^14^C-MeAIB transfer were both reduced in either sex, even after accounting for the reduced surface area of the Lz, with evidence for a more severe impact in female *Igf2*^EpiKO^ mutants. The key determinants governing glucose transfer across the placenta are the trans-placental gradient and the amount of glucose utilized by the placenta to meet its own metabolic needs (Henriksen et al., 2022). No indications were found to support alterations in the trans-placental glucose gradient, a conclusion corroborated by the similarity in blood glucose concentrations between *Igf2*^EpiKO^ mutant and control fetuses at E18.5. Furthermore, there were no discernible differences in the amount of glycogen stored in placenta or liver at E18.5. However, at both E15.5 and E18.5 mRNA levels of all three glucose transporter genes investigated in this study (i.e. *Slc2a1*, *Slc2a3* and *Slc2a8*) were reduced in placenta Lz samples of female *Igf2*^EpiKO^ mutants. The reduction in the amount of ^14^C-MeAIB transported by placenta at E18.5 after accounting for the diminished Lz surface area was even more striking than that of ^3^H-MeG and, once again, was more severe in female *Igf2*^EpiKO^ mutants. Consistent with the *in vivo* data, mRNA levels of one of the three genes encoding system A amino acid transporters (*Slc38a2*) were reduced within the placenta Lz at E18.5, with an additional tendency for reduced *Slc38a1* mRNA levels, suggesting that their reduced abundance is responsible for the effects observed. However, further investigations are necessary to elucidate the precise mechanisms underlying the reduced expression of these transporter genes. Consistent with the current data, we have previously uncovered a positive linear relationship between placental clearance of glucose and the body weight of fetuses at E15.5, and between placental clearance of MeAIB and fetal weight at E18.5 (Coan et al., 2011). It is also noteworthy that glucose and system A amino acid transporter genes are established mediators of placental functional adaptations to meet fetal growth requirements, both in response to genetic manipulations and altered environmental conditions *in utero* (Coan et al., 2008b, 2010; Constância et al., 2005; Angiolini et al., 2011; Sferruzzi-Perri et al., 2011, 2023).

Third, we observed compelling evidence pointing towards a failure of placental adaptations at the end of gestation, i.e. when normally – in absolute terms − the fetus grows most rapidly. By analyzing growth distribution curves and selecting criteria used to define SGA and FGR in humans, we found that the growth restriction of *Igf2*^EpiKO^ mutant fetuses, which becomes more severe with advancing pregnancy, is followed by placental growth restriction in a large fraction of conceptuses. These patterns of failed feto-placental growth were more severe in female *Igf2*^EpiKO^ mutants. This progressive feto-placental growth restriction was associated with impaired functional adaptations of the placenta near term, particularly with a reduction in the placental transfer of ^14^C-MeAIB within *Igf2*^EpiKO^ mutant fetuses at E18.5. In both genotypes, ^3^H-MeG per gram fetus decreased between E15.5 and E18.5, a gestational change that is likely to be attributable to the improved maternal insulin sensitivity near term, which – in turn –reduces the trans-placental glucose gradient (Musial et al., 2016). By contrast, over the same period of late-gestation we observed a dramatic increase in the uptake of ^14^C-MeAIB per gram of fetus, irrespective of genotype. Based on these observations, we can infer that the reduction in ^14^C-MeAIB uptake per gram of fetus in *Igf2*^EpiKO^ mutants at E18.5 directly contributes to the observed FGR, underscoring the preeminent role of amino acids in sustaining fetal growth near term (Coan et al., 2011). This failure in adaptation at the end of the gestation also appears to be closely associated with changes in the characteristics of UA blood flow. Consistent with previous observations in mouse (Mu and Adamson, 2006), our findings indicated a physiological increase in PSV and EDV as pregnancy progressed, accompanied by a decrease in the RI. However, although no signs of altered blood flow through the smaller UA were evident at E15.5, we noted a significant increase in umbilical artery PI (UAPI) in mutant fetuses at E18.5, associated with a severe reduction of blood flow through the UA. This observation is relevant for human pregnancy, as most cases of FGR are clinically diagnosed at later gestational age (Chew and Verma, 2023).

Previous murine studies of FGR in the context of placental dysfunction and altered transcription have demonstrated abnormal UA flow patterns in later pregnancy, including an increased PSV (Shreeve et al., 2021). Drawing parallels from human observations, the alterations in UA blood flow characteristics observed at E18.5 in *Igf2*^EpiKO^ mutants are significant. Ultrasound Doppler assessment of the UAPI is the primary surveillance tool for FGR in human pregnancy (Royal College of Obstetricians and Gynaecologists Green-top Guideline No. 31, Morris et al., 2024), where monitoring using this method allows planning of early delivery to reduce perinatal mortality. The UAPI serves as a surrogate marker for the fetal well-being and can reflect vascular impedance within the feto-placental circulation (Mone et al., 2016), with a raised PI often being a late manifestation of chronic placental dysfunction/insufficiency. In the context of FGR, UAPI changes demonstrate a stepwise pattern of decline for which a raised PI is usually followed by absent and/or reversed end-diastolic flow. Therefore, isolated rises in UAPI, even with positive end-diastolic flow, can be sufficient to indicate delivery in the interests of the fetus.

Recent mathematical models have suggested a significant impact of UA blood flow on the transfer of solutes across the placental interchange barrier (Jensen and Chernyavsky, 2019), and reduced velocity has been predicted to have a negative effect on the amount of facilitative amino acids, such as alanine (Panitchob et al., 2016), reaching the fetus. Alanine is a recognized substrate of the system A amino acid transporters (Johnson and Smith, 1988) investigated in our study. However, consistent with the findings of our current study, in human pregnancies complicated by FGR, the reduced UA blood flow has been associated with a fetal glucose delivery at term, which is proportionate to fetal size (Cetin et al., 2020). Cetin and colleagues also reported a reduction of umbilical oxygen delivery to the fetus, a key constituent required for fetal growth, even when data were normalized per fetal body weight (Cetin et al., 2020). Together, these studies support a significant role for the reduced blood flow within the UA in the pathogenesis of FGR.

Our findings in the *Igf2*^EpiKO^ mouse model hold notable relevance for human pregnancies complicated by FGR. Abnormal Doppler findings in the UA serve as key criteria for distinguishing between SGA and FGR, as well as for discerning between early- and late-onset FGR (Sovio et al., 2015; Mylrea-Foley et al., 2023). Additionally, previous research conducted on microvillous plasma membrane vesicles isolated from placentae that had been associated with SGA and FGR has revealed diminished activity of system A amino acid transporters (per milligram of placental protein), particularly in groups affected by severe FGR (Glazier et al., 1997; Harrington et al., 1999; Jansson et al., 2002; Shibata et al., 2008; McIntyre et al., 2020). Drawing parallels between the observations made in our study and these prior findings in humans, we propose that *Igf2*^EpiKO^ mice are pertinent to model FGR in humans. In this current study, we used genetic engineering to specifically reduce fetal demand for growth and analyze the impact on placental adaptations. However, similar placental adaptive mechanisms could take place in human pregnancies associated with the mutation of growth-promoting genes that are only expressed in the fetus but silent, i.e. not transcribed, in the placenta.

Growing evidence for the key role played by the placenta in the pathophysiology of FGR has led recently to the development of placenta-targeted therapies, including gene therapy protocols, stem-cell-based therapies and drug-delivery strategies, aimed to correct fetal growth (Krishnan and David, 2017; Davenport et al., 2022; Cui et al., 2024). Our findings suggest that therapy methods enhancing placental IGF2 availability during later stages of pregnancy, when a fully functional placenta is key to supporting a rapidly growing fetus, are an attractive potential strategy for improving outcomes in pregnancies associated with FGR. In support of this idea, a previous study found that selective delivery of IGF2-loaded nanoparticles targeting the placenta of *Igf2* P0^+/−^ FGR model mice between E11.5 and E15.5 improves fetal growth (King et al., 2016). Likewise, continuous delivery between E12.5 and E18.5 of the IGF2 analogue IGF2^Leu27^ – which binds exclusively to the IGF2R receptor – via minipumps leads to a partial rescue of fetal growth in the eNOS^−/−^ mouse model of FGR (Charnock et al., 2016).

Our study, however, has several notable limitations. The sample size was limited for the *in vivo* studies (placental transfer assays and ultrasonography), as well as for histological (placental stereology and placental and liver glycogen staining) and some biochemical assays (measurement of liver and placental glycogen content). Although we explored the role of system A amino acid transporters – which has been strongly linked with fetal growth in previous studies using both human and animal models (Sandovici et al., 2012; Vaughan et al., 2017) – due to lack of specific non-metabolizable substrates we did not assess all other placental transporters that mediate the transfer of essential (system L), neutral (ASC, B0, N, Gly), cationic (systems y^+^, y^+^L, b^0,+^) or anionic (system X^−^_AG_) amino acids (Vaughan et al., 2017). However, the finding that mRNA levels of two additional amino acid transporter genes (*Slc1a5* and *Slc7a2*) were also downregulated at E15.5 and E18.5 demonstrates that amino acid transport impairment is not restricted to the system A amino acid transporters in the *Igf2*^EpiKO^ model. Additionally, we did not investigate directly placental passive permeability. Nevertheless, it is well established that theoretical diffusion capacity (TDC) serves as a reliable predictor of placental passive permeability capacity (Sibley et al., 2004). Based on the reduction of TDC observed at both E15.5 and E18.5, we were able to anticipate a reduced trans-placental flux of H_2_O and other solutes transferred by passive diffusion, which might contribute directly to the reduced fetal growth, as recently proposed (Angiolini et al., 2021). Another parameter not investigated in this study was the impact of placental morphological and UA blood flow alterations on the gaseous exchange between mother and fetus, known to contribute to fetal growth (Higgins et al., 2016; Cetin et al., 2020; Moore, 2021). Our observation that the glucose transporter genes were differentially expressed at E15.5 and E18.5, despite a largely normal trans-placental glucose transfer require further investigation. In rodents, GLUT1 is located on all placental membranes, whereas GLUT3 is found specifically on the maternal surface of the labyrinthine trophoblast (Zhou and Bondy, 1993). It will be interesting to assess whether *Igf2*^EpiKO^ mutants show changes in the normal distribution of these two glucose transporters on placental membranes. Another significant limitation in our study was the necessity to use general anesthetic for the mice to perform *in vivo* measurements, such as ultrasonography and placental transfer assays. By contrast, sonographic measurements during human pregnancy are performed while patients are conscious and at rest. The anesthetic agents used in our study, i.e. intraperitoneal injections of fentanyl–fluanisone–midazolam (FFM) led to recordings – such as fetal heart rate, UA velocity and UA blood flow – that are higher than those reported in previous studies performed in mice anesthetized using isoflurane (Rahman et al., 2017; Cahill et al., 2019). This is consistent with previous observations showing higher heart rates in rats that had been anesthetized with FFM compared to isoflurane (Secher et al., 2017). The higher intra-observer and inter-observer coefficients of variation for UA wall thickness and EDV measurements – particularly at E15.5 (Table S2) – imply caution in interpreting these results. Last, although we recorded fetal sex in all measurements performed in the study, the smaller sample sizes used in some analyses, such as placental stereology and ultrasonography, precluded us from consistently assessing the impact of this, potentially important, parameter. In the case of placental stereology, we included equal numbers of male and female samples. However, in the case of ultrasonography, achieving a perfect balance between the two sexes was impossible, owing to the nature of analysis, and observers being unaware of genotype and fetal sex. Despite these noteworthy limitations, we observed a more severe impact on fetal and placental growth, as well as on trans-placental transfer of glucose and system A amino acids in female *Igf2*^EpiKO^ mutants. There is already evidence for sex-specific differences in the abundance of IGF2 receptors and downstream signaling pathways in the mouse placenta (Aykroyd et al., 2022). However, uncovering the mechanisms that mediate the interaction between IGF2 signaling and fetal sex warrants future investigation.

In conclusion, our study provides compelling experimental evidence that, in the face of a reduced genetic demand for growth, the total capacity for placental nutrient transfer is blunted towards the end of gestation in the *Igf2*^EpiKO^ mutant. This appears to be largely due to a reduced interhemal interchange surface area of the placental Lz but, probably, also reflects a contribution from the disproportionately reduced placental abundance of specific amino acid and glucose transporters, and altered patterns of umbilical blood flow. These findings uncover novel and potentially targetable adaptive mechanisms within the placenta, which could improve fetal growth, and ameliorate the perinatal and long-term outcomes in pregnancies complicated by FGR.

## MATERIALS AND METHODS

### Mice

Mice were bred, maintained and mated under pathogen-free conditions at the University of Cambridge Phenomics Unit (West Forvie Site), in accordance with the University of Cambridge Animal Welfare and Ethical Review Body (AWERB) and the United Kingdom Home Office Regulations. Mice were fed a standard chow diet (SDS, Essex, UK) and housed (2-5 mice per cage) in a temperature-controlled room (22°C), with a 12-h light-dark cycle. Food and water were available *ad libitum*. All *in vivo* experiments (placental transfer assays, ultrasound imaging) were performed in the morning, starting at around 9 am and followed immediately by killing using a schedule 1 method (https://www.gov.uk/guidance/guidance-on-the-operation-of-the-animals-scientific-procedures-act-1986). Tissue collections for histological, biochemical or molecular analysis were also performed in the morning following a schedule 1 method. Whenever possible, we applied the 3R principles (i.e. replacement, reduction, refinement; Prescott and Lidster, 2017) to maximize the usage of samples and minimize the number of mice used. However, we did not perform more than one *in vivo* assay per mouse, and we did not use any tissue for histology or qRT-PCR measurements following an *in vivo* assay to avoid any effect related to anesthesia.

The *Igf2*^fl/fl^ mice were generated in our laboratory (Hammerle et al., 2020) and the *Meox2*^Cre^ mice (Tallquist and Soriano, 2000) were imported from the Jackson Laboratory (Maine, USA). *Meox2*^Cre^ is active starting at E4.5 in the epiblast, which gives rise to the entire embryo proper, as well as the feto-placental capillaries (Tallquist and Soriano, 2000). Both lines were bred into an inbred C57BL/6J genetic background for >10 generations prior to this work. For this study, we timed mated young adult (age 6-12 weeks) homozygous *Igf2*^fl/fl^ male mice with young (age 6-12 weeks) virgin heterozygous *Meox2*^Cre^ female mice to generate litters with a half of the conceptuses being mutants (*Meox2*^Cre/+^; *Igf2*^+/fl^, referred to as *Igf2*^EpiKO^) and half being littermate controls (*Meox2*^+/+^; *Igf2*^+/fl^, referred to as C). The morning of discovering the copulation plug was counted as embryonic day 0.5 (E0.5). Genotyping was performed using standard PCR with DNA extracted from ear biopsies (adult mice) or tails (fetuses), Red Taq Ready PCR system (Sigma) and previously published primers (Sandovici et al., 2022), followed by separation of PCR amplicons by using agarose gel electrophoresis.

### Placenta stereology

Placenta stereology analysis was performed as previously described (Coan et al., 2004; Sandovici et al., 2022). Histology was performed in *n*=6 control (C) and *n*=6 *Igf2*^EpiKO^ mutants from *N*=3 litters, at each developmental stage (E13.5, E15.5 and E18.5). Formulas used to calculate the labyrinthine zone surface area, the harmonic mean membrane thickness (HMT) and the theoretical diffusion capacity (TDC) have been described previously (Coan et al., 2004).

### Placental transfer assays

Placental transfer assays were performed as described before (Constância et al., 2005). Briefly, pregnant mice at E15.5 or E18.5 were anesthetized with an intraperitoneal injection of 0.4 ml fentanyl:fluanisone:midazolam (1:1:2) solution in water (FFM) (Janseen Animal Health). A neck incision was made, and the jugular vein was exposed. A 200-μl bolus of PBS containing 3.5 μCi (0.13 MBq) of ^3^H-methyl-D-glucose (^3^H-MeG; NEN NEC-377, 2.1 GBq/mmol; Perkin Elmer) and/or ^14^C-methylaminoisobutyric acid (^14^C-MeAIB; NEN NEC-377, 2.1 GBq/mmol; Perkin Elmer) was then injected into the jugular vein via a short length of tubing attached to a 27-gauge needle and connected to a 1-ml syringe. Between 2 and 2.5 min after injection of tracer, animals were killed, and conceptuses dissected by hysterectomy. ^3^H-MeG data were collected in *N*=5 litters at E15.5 (*n*=22 C and *n*=22 *Igf2*^EpiKO^ mutants) and *N*=7 litters at E18.5 (*n*=30 C and *n*=29 *Igf2*^EpiKO^ mutants). ^14^C-MeAIB data were collected in *N*=5 litters at E15.5 (*n*=22 C and *n*=22 *Igf2*^EpiKO^ mutants) and *N*=5 litters at E18.5 (*n*=22 C and *n*=15 *Igf2*^EpiKO^ mutants). Fetuses were lysed for 1 week at 55°C in 2 ml (E15.5 fetuses) or 4 ml (E18.5 fetuses) of Biosol (National Diagnostics). Aliquots of fetal samples (250 µl for E15.5 and 500 µl for E18.5) were then added to 4 ml scintillation fluid for beta counting (Packard Tri-Carb 1900). Measurements were performed in duplicate for each fetal homogenate. Radioactive counts in each fetus, i.e. disintegrations per minute (DPM), were then used to calculate the amount of radioisotope transferred per gram fetus or per mm^2^ of interhemal surface area. Average values for control and mutant fetuses within a litter were then calculated and used for statistical analyses. The fetal accumulation of radioisotope per mm^2^ surface area gives a relative measure of placental transfer of the solute. The fetal accumulation of radioisotope expressed relative to fetal weight gives a relative measure of the amount of solute received by the fetus.

### Fetal glucose

Fetal glucose measurements were recorded at E18.5, immediately after decapitation, using a glucose meter and test strips (AlphaTRAK). Data were collected from *N*=14 litters. To minimize variability due the lag between death of the mother and killing of the fetuses, only measurements in the first fetus of each genotype (one fetus per litter) were used for statistical analysis.

### Glycogen content

The glycogen content was measured in the liver and whole placenta collected from E18.5 litters, using enzymatic degradation of glycogen stores into glucose, followed by glucose measurement, as previously published (Franko et al., 2007). Briefly, tissue homogenates were prepared in cold deionized H_2_O (100 mg/ml); then 100 μl aliquots of homogenate were incubated with the *Aspergillus niger* enzyme amyloglucosidase (#A1602; Sigma) at 55°C using appropriate buffer conditions. The samples were then deproteinized using zinc sulphate and barium hydroxide, after which the mixture was centrifuged, and the supernatants were collected and stored at −20°C until glucose measurements were performed at the Core Biochemical Assay Laboratory (CBAL; Addenbrooke's Hospital, Cambridge, UK). Glycogen measurements of the liver were performed in samples collected from *N*=4 litters (*n*=17 C and *n*=18 *Igf2*^EpiKO^ mutants). Glycogen measurements of placenta were performed in *N*=2 litters (*n*=8 C and *n*=9 *Igf2*^EpiKO^ mutants). Each tissue sample was incubated in duplicate, with and without amyloglucosidase. Glucose concentration obtained in absence of enzyme (tissue blank) was subtracted from the value obtained in the presence of enzyme.

### Periodic acid−Schiff staining

Periodic acid−Schiff (PAS) staining was performed on paraffin sections of E18.5 livers and placentae (*n*=6 per genotype from a total of *N*=3 litters), as described previously (Coan et al., 2006). Briefly, placentae were first hemisected, and both livers and placentae were fixed in 10% formalin overnight, and then embedded in paraffin. The paraffin blocks were sectioned at 5 μm thickness using a rocking microtome. Sections were then deparaffinized and hydrated, stained with 0.5% PAS solution (#1004821000, Sigma) and Schiff reagent (#1090330500, Sigma), dehydrated and then mounted with coverslips. Negative controls were prepared by placing the slides in warm diastase solution (#1036040050, Sigma) for 15 min before the PAS staining.

### Real time qRT-PCR analysis

Placenta Lz was dissected under a microscope, flash-frozen in liquid nitrogen and stored at −80°C until use. Total RNA was extracted using RNeasy Plus Mini Kits (#74134, Qiagen) from placenta Lz samples of female conceptuses selected from *N*=3 litters (*n*=6 C and *n*=7 *Igf2*^EpiKO^ mutants at E13.5, *n*=8 C and *n*=7 *Igf2*^EpiKO^ mutants at E15.5 and *n*=8 C and *n*=7 *Igf2*^EpiKO^ mutants at E18.5); RNA concentrations were measured using a NanoDrop spectrophotometer (Thermo Fisher Scientific) and quality was assessed using agarose gel electrophoresis. Reverse transcription to obtain cDNA was performed using the RevertAid RT Reverse Transcription Kit (#K1622, Thermo Fisher Scientific) and RT(−) reactions were used as negative control. Real-time quantitative reverse transcription PCR (qRT-PCR) was performed in triplicate, with the SYBR Green JumpStart Taq Ready Mix (#S4438, Sigma-Aldrich) and custom-made primers (Table S1), using an ABI Prism 7900 system (Applied Biosystems). Normalization of gene expression was performed against three housekeeping genes (*Gapdh*, *Pmm1*, *Sdha*) that had been detected to be stably expressed in mutants and controls, and across the E13.5-E18.5 developmental time window (Sandovici et al., 2022). Relative expression levels were calculated using the 2^−ΔΔCt^ method (Livak and Schmittgen, 2001).

### Ultrasound imaging

At E15.5 or E18.5, pregnant mice were anesthetized by intraperitoneal injection of 0.4 ml FFM and their fur was removed from the anterior abdominal region. Ultrasound imaging was performed in *N*=3 litters at E15.5 (*n*=7 C and *n*=8 *Igf2*^EpiKO^ mutants) and *N*=3 litters at E18.5 (*n*=7 C and *n*=9 *Igf2*^EpiKO^ mutants), without any prior knowledge of the fetal genotypes, by using the Vevo2100 Imaging System (VisualSonics, Canada) fitted with a MS 550D transducer (Zhou et al., 2002). Electrocardiography, respiratory rate and core temperature monitoring were performed continuously throughout the procedure. Pre-warmed gel for sonography was used as a coupling medium. Stage temperature was adjusted, to maintain a core temperature of 37°C. UA inner and outer diameters were measured in M-Mode at a frequency of 40 MHz, with the ultrasound beam perpendicular to the vessel. UA blood flow PSV and EDV were measured in pulsed-wave (PW) Doppler Mode at 32 MHz frequency and the angle of insonation, i.e. the angle of the ultrasound beam relative to the flow direction was <30°. Measurements were acquired from up to six individual conceptuses per litter, with two or three fetuses from each uterine horn, closest to the midline, whose positions within each uterine horn were mapped during the ultrasound measurements with high confidence. A sequence of several recordings, i.e. cineloops, of up to 5 s each, of M-mode and Power Doppler spectrum were saved for each fetus, and used offline for later quality assessment and measurements. Immediately after the ultrasound measurements, pregnant mice were killed, fetal positions confirmed and their genotypes verified by PCR.

Prior to performing measurements, recorded data were assessed for quality. For M-mode recordings, we excluded those affected by maternal gasping or fetal movement. For Power Doppler recordings, only recordings of strong contrast, highest amplitude and sharp contour were considered optimal, and included in the measurements. These quality checks led to the exclusion of three out of 30 fetuses (10%) included in the study, with data analysis being performed in *n*=6 C and *n*=7 *Igf2*^EpiKO^ mutants at E15.5 and *n*=6 C and *n*=9 *Igf2*^EpiKO^ mutants at E18.5. Data analysis was performed by two independent observers over three cardiac cycles and the results were averaged. For each parameter measured at the two developmental time points (E15.5 and E18.5) we calculated the intra-observer and inter-observer coefficients of variation (Table S2) as previously reported (Blanca et al., 2023). Only two parameters, i.e. EDV and wall thickness of the UA at E15.5, did associate coefficient of variation values that were >10% but <15%. This would classify these values as being moderately repeatable and all other parameters being highly repeatable (see Table S2 for intra-observer and inter-observer coefficients of variation). Pulsatility index (PI) was calculated using the equation PI=(PSV-EDV)/(UA mean velocity) and resistivity index (RI) was calculated by using the equation RI=(PSV-EDV)/PSV. We also measured the velocity-time integral (VTI) and calculated the UA inner area and the fetal heart rate. Using on these parameters, UA blood flow was calculated by applying the formula: UA blood flow (ml/min)=[VTI (mm)×UA inner area (mm2)×heart rate (bpm)]/1000, as previously published (Zhou et al., 2014).

### Statistical analysis

All statistical analyses were performed using GraphPad Prism 10. For each experiment, sample size calculations were carried out based upon similar studies performed by our own group (Constância et al., 2005; Shreeve et al., 2021; Sandovici et al., 2022). For analyses of fetus and placenta growth, weights were first organized in bins, according to their frequency distribution, with a least-squares fit. Then, a non-linear, Gaussian type line was fitted, according to the 95% confidence interval (CI). For statistical analyses between two groups, to determine whether the data were of a Gaussian distribution, the Shapiro-Wilk test was first applied, followed by Mann–Whitney tests or two-tailed unpaired Student's *t*-tests with Welch's correction, as appropriate. Statistical significance between two groups across two or three developmental stages was performed using two-way ANOVA tests. In the case of placental transfer assays, to account for differences in the exposure time to the radiolabels between litters, repeated measures two-way ANOVA tests were applied instead, which allow pairing the average values calculated within a litter for each of the two genotypes. Two-way ANOVA tests were followed by Sidak's multiple comparisons tests. The numbers of samples (*n*) or litters (*N*) used for each experiment are indicated in figure legends.

## Supplementary Material

10.1242/dmm.050719_sup1 Supplementary information
