# Supplementary material for: A genetically small fetus impairs placental adaptations near term
Source: Dis Model Mech. 2024 Aug 29;17(8):dmm050719. doi: 10.1242/dmm.050719 (PMC11381921; doi:10.1242/dmm.050719)
Supplement: Supplementary information [file dmm-17-050719-s1.pdf]

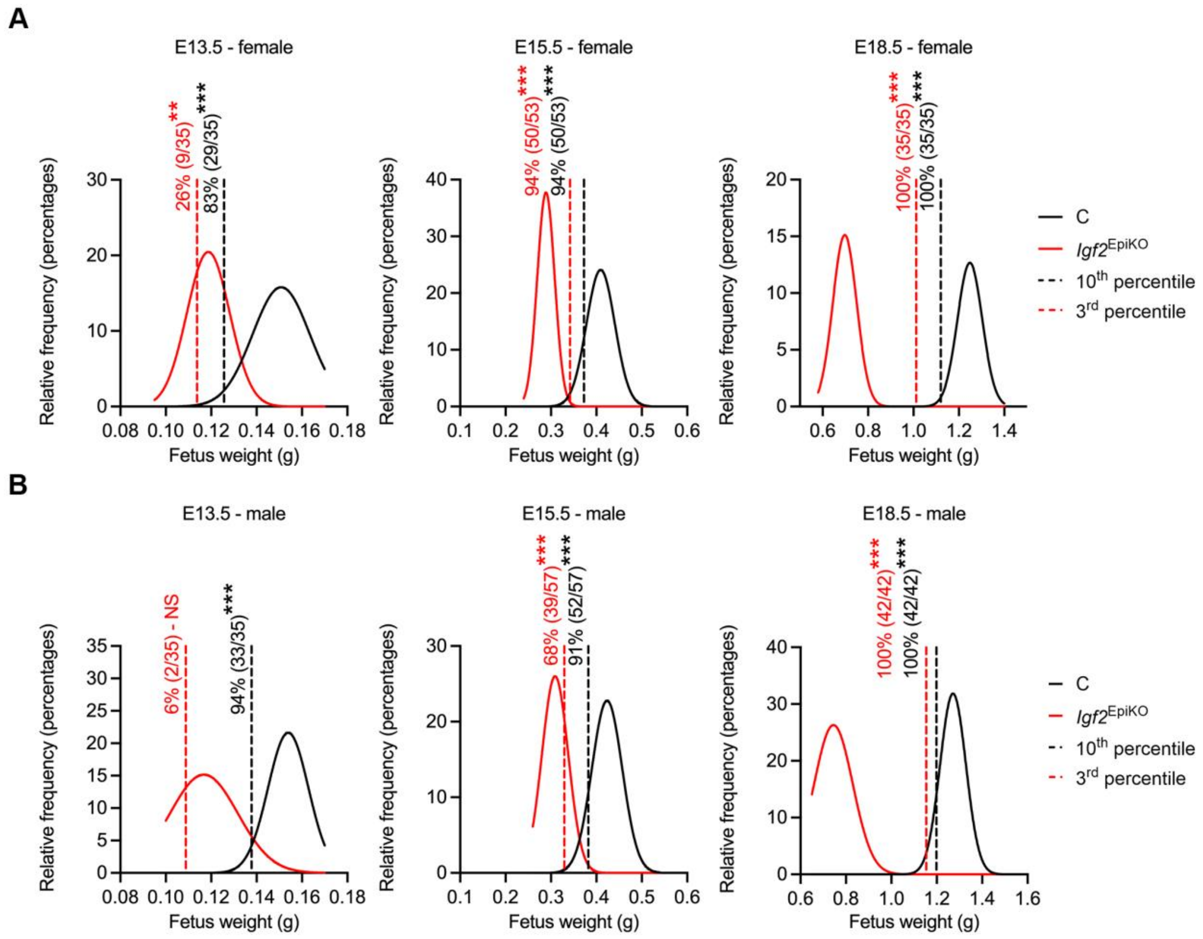


**Fig. S1. Fetal weight distribution curves for *Igf2*$^{EpiKO}$ mutants versus littermate controls.** (A) Fetal weight distribution curves for female *Igf2*$^{EpiKO}$ mutants versus littermate controls. The distribution curves were generated from N = 16 litters at E13.5 (n = 41 C and n = 35 *Igf2*$^{EpiKO}$), N = 28 litters at E15.5 (n = 56 C and n = 53 *Igf2*$^{EpiKO}$) and N = 18 litters at E18.5 (n = 35 C and n = 35 *Igf2*$^{EpiKO}$). (B) Fetal weight distribution curves for male *Igf2*$^{EpiKO}$ mutants versus littermate controls. The distribution curves were generated from N = 16 litters at E13.5 (n = 33 C and n = 35 *Igf2*$^{EpiKO}$), N = 28 litters at E15.5 (n = 61 C and n = 57 *Igf2*$^{EpiKO}$) and N = 18 litters at E18.5 (n = 33 C and n = 42 *Igf2*$^{EpiKO}$). To apply a best-fit Gaussian line, fetal weights were first organised in bins, according to their frequency distribution, with least squares fit. For all panels, the black and the red dashed lines represent the 10$^{th}$ and the 3$^{rd}$ percentile of the C curves, respectively; the % values indicate the fractions of fetuses that fall below these thresholds, with absolute numbers provided between parentheses; NS – non-significant; ** P < 0.01; *** P < 0.001 by Fisher's exact tests.

Disease Models & Mechanisms • Supplementary information

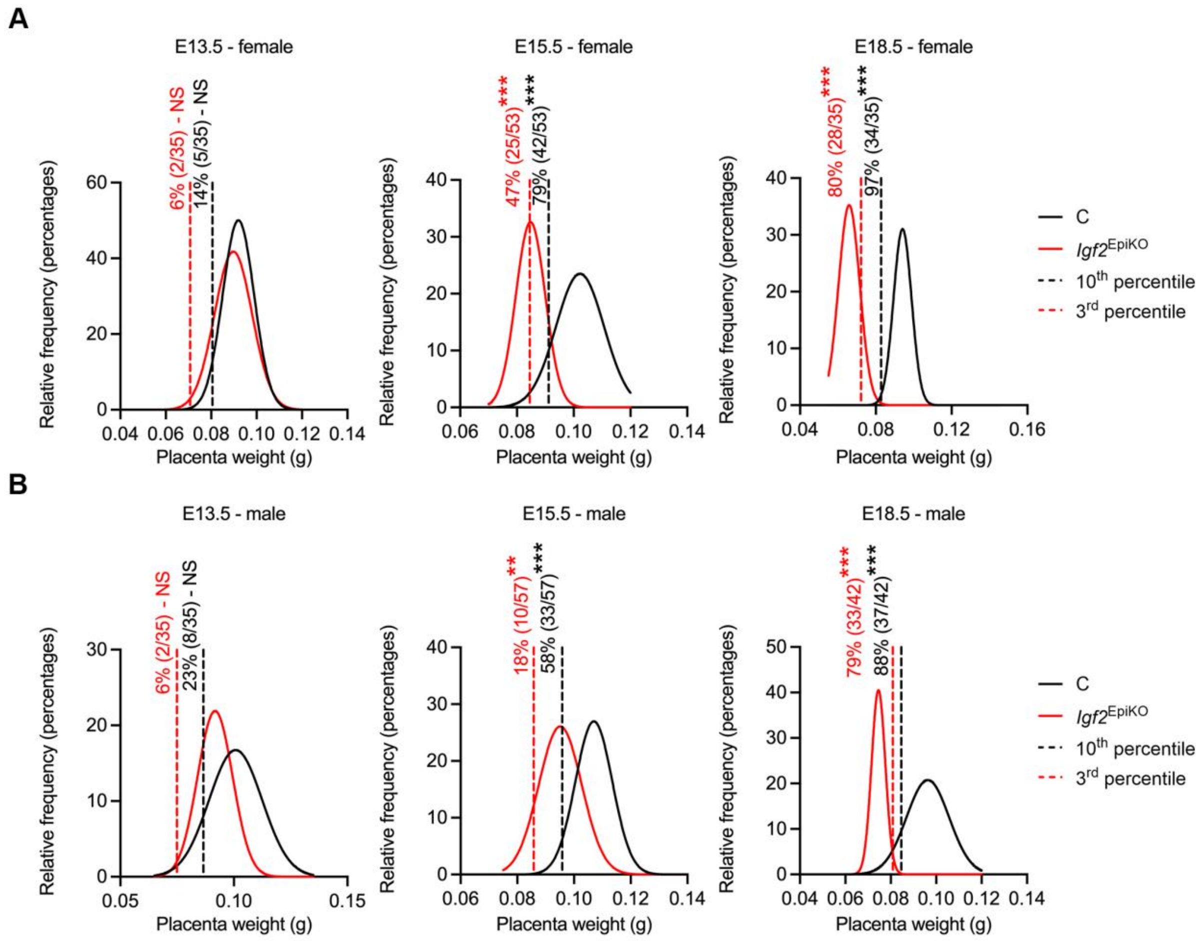


**Fig. S2. Placental weight distribution curves for *Igf2*$^{EpiKO}$ mutants versus littermate controls.** (A) Placental weight distribution curves for female *Igf2*$^{EpiKO}$ mutants versus littermate controls. The distribution curves were generated from N = 16 litters at E13.5 (n = 41 C and n = 35 *Igf2*$^{EpiKO}$), N = 28 litters at E15.5 (n = 56 C and n = 53 *Igf2*$^{EpiKO}$) and N = 18 litters at E18.5 (n = 35 C and n = 35 *Igf2*$^{EpiKO}$). (B) Placental weight distribution curves for male *Igf2*$^{EpiKO}$ mutants versus littermate controls. The distribution curves were generated from N = 16 litters at E13.5 (n = 33 C and n = 35 *Igf2*$^{EpiKO}$), N = 28 litters at E15.5 (n = 61 C and n = 57 *Igf2*$^{EpiKO}$) and N = 18 litters at E18.5 (n = 33 C and n = 42 *Igf2*$^{EpiKO}$). To apply a best-fit Gaussian line, placental weights were first organised in bins, according to their frequency distribution, with least squares fit. For all panels, the black and the red dashed lines represent the 10$^{th}$ and the 3$^{rd}$ percentile of the C curves, respectively; the % values indicate the fractions of placentae that fall below these thresholds, with absolute numbers provided between parentheses; NS – non-significant; ** P < 0.01; *** P < 0.001 by Fisher's exact tests.

Disease Models & Mechanisms • Supplementary information

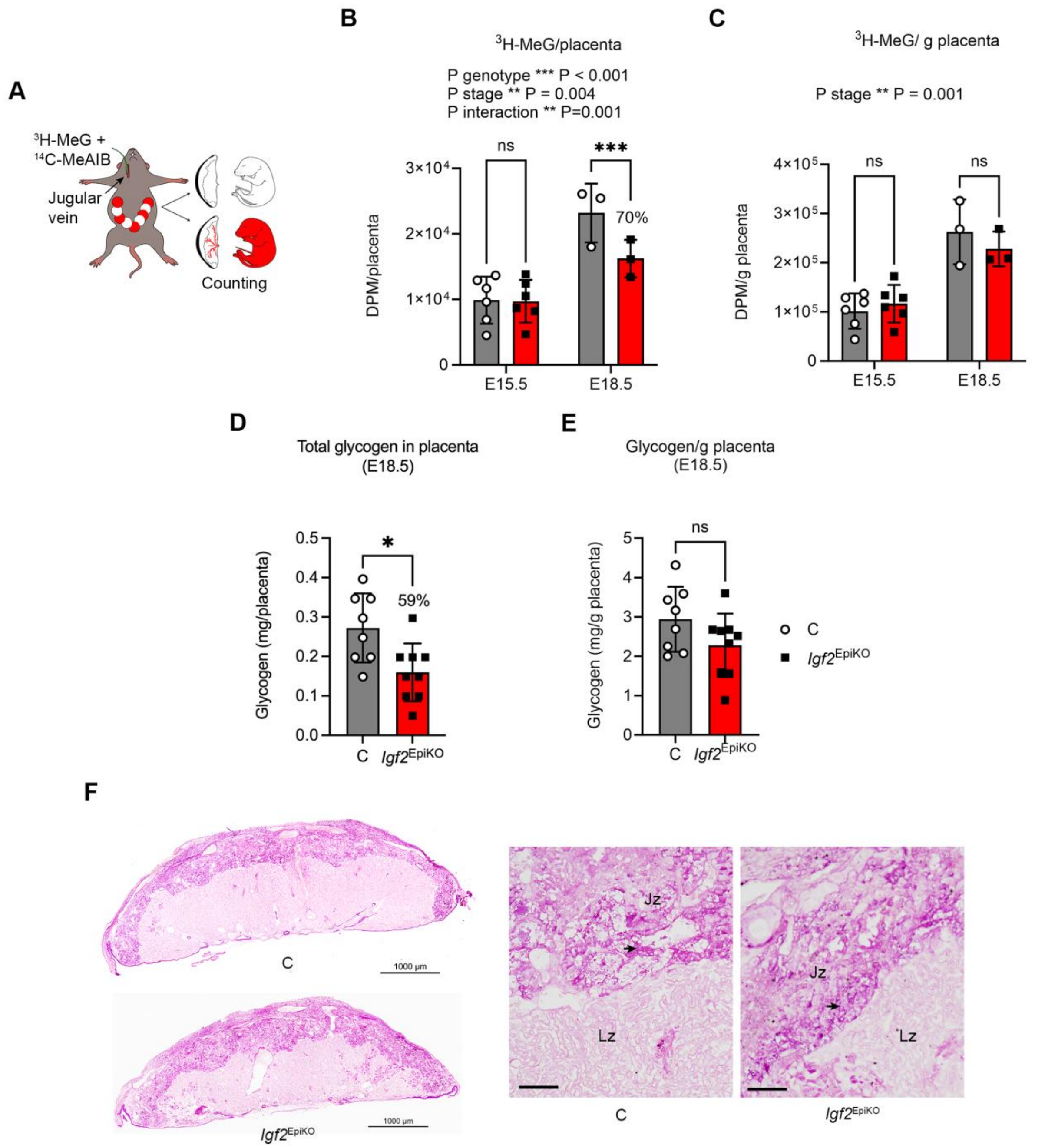


**Fig. S3. Placental accumulation of $^{3}$H-methyl-D-glucose ($^{3}$H-MeG) and glycogen.** (A) Diagram depicting the principle of placental transfer assays: pregnant females under anaesthesia receive a fixed dose of radioactively-labelled tracer through the jugular vein; after an interval of 2' – 2'30", the female is killed the fetuses and placentae are dissected and used for counting. Measurements of disintegrations per minute (DPM) in placenta, represented as total counts per placenta (B) or counts per gram of placenta (C). For both graphs, data was collected in N = 6 litters at E15.5 (n = 28 C and n = 26 *Igf2*$^{EpiKO}$ mutants) and N = 3 litters at E18.5 (n = 19 C and n = 7 *Igf2*$^{EpiKO}$ mutants). (D) Quantification of glycogen deposits in placenta at E18.5 and represented as total amounts per placenta. (E) Quantification of glycogen deposits in placenta at E18.5 and normalized per unit of

Disease Models & Mechanisms • Supplementary information

placenta weight. Data in panels (D) and (E) was collected in N = 2 litters with n = 8 C and n = 9 *Igf2*$^{EpiKO}$ mutants. (F) Representative PAS stains for glycogen content in placentae at E18.5 confirming similar patterns of glycogen deposits in the two genotypes. Images on the right side depict high-magnifications obtained in the images provided in the left side. For the low-magnification images, the sizes of each scale are indicated in the figures; for the high-magnification images, scale bars are 100 µm. Jz – junctional zone, Lz – labyrinthine zone, arrows point to glycogen deposits (dark purple). Data is shown as average values per litter, with mean values ± SD (B – C) or individual values, with mean values ± SD (D – E). P values shown above the graphs correspond to statistically significant two-way ANOVA tests; ns – non-significant, * – $P < 0.05$; *** – $P < 0.001$ calculated by Sidak's multiple comparison tests following two-way ANOVAs (B – C) or student t tests with Welch's correction (D – E); % values indicate *Igf2*$^{EpiKO}$/C ratios.

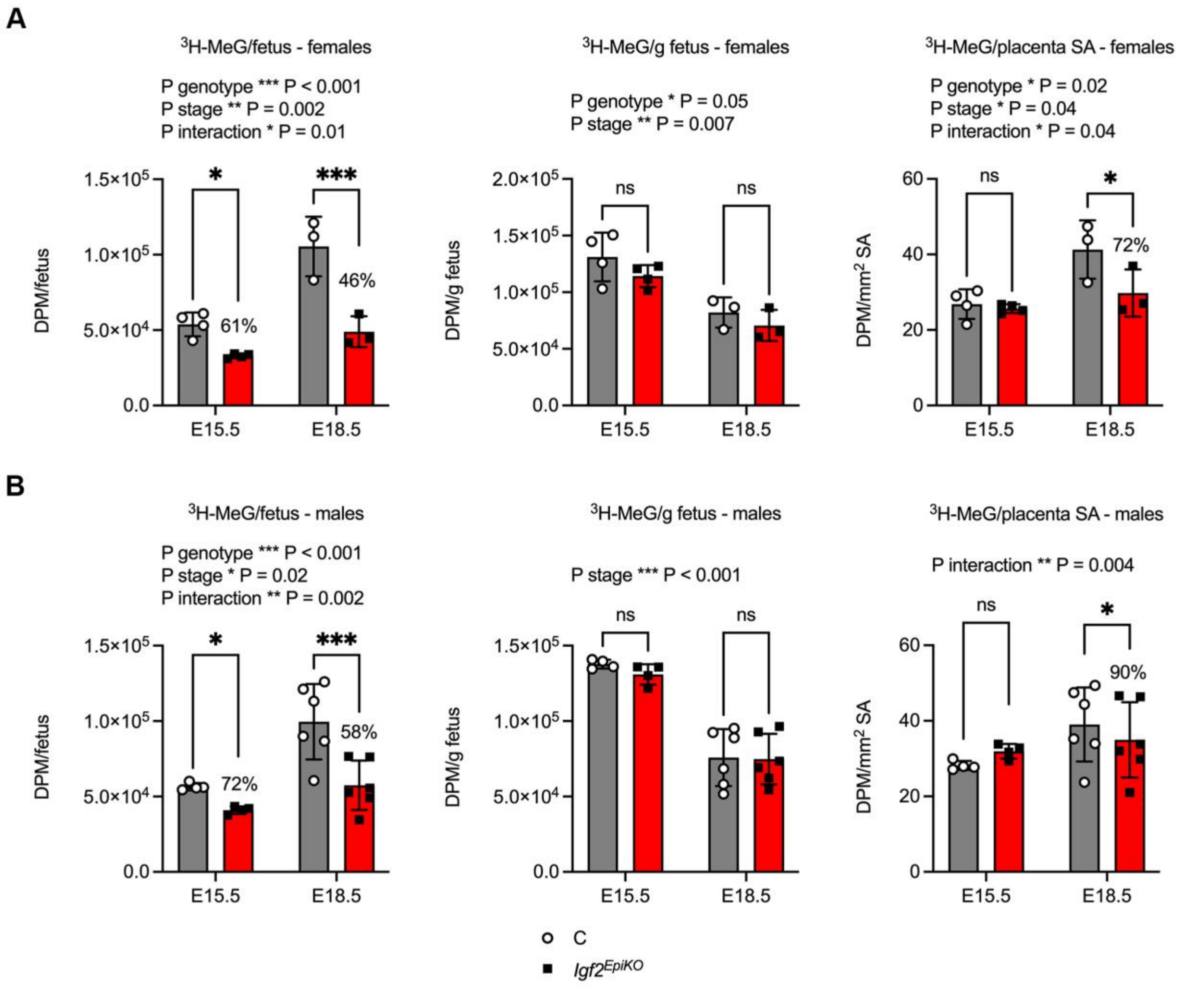


**Fig. S4. Analysis of placental transfer of $^3$H-methyl-D-glucose ($^3$H-MeG) in relation to fetal sex.** (A) Measurements of disintegrations per minute (DPM) in the female fetuses, represented as counts per fetus (left panel), counts per gram fetal weight (middle panel) and counts per mm$^2$ labyrinthine zone surface area – SA (right panel). Data in (A) was collected in N = 4 litters at E15.5 (n = 9 C and n = 13 *Igf2*$^{\mathrm{EpiKO}}$ mutants) and N = 3 litters at E18.5 (n = 8 C and n = 8 *Igf2*$^{\mathrm{EpiKO}}$ mutants). (B) Measurements of disintegrations per minute (DPM) in the male fetuses, represented as counts per fetus (left panel), counts per gram fetal weight (middle panel) and counts per mm$^2$ labyrinthine zone surface area – SA (right panel). Data in (B) was collected in N = 4 litters at E15.5 (n = 11 C and n = 6 *Igf2*$^{\mathrm{EpiKO}}$ mutants) and N = 6 litters at E18.5 (n = 16 C and n = 18 *Igf2*$^{\mathrm{EpiKO}}$ mutants). For all graphs, data is presented as average values per litter, with mean values ± SD. P values shown above the graphs correspond to statistically significant repeated measures two-way ANOVA tests; ns – non-significant, * – P < 0.05, *** – P < 0.001 calculated by Sidak's multiple comparison tests following repeated measures two-way ANOVAs; % values indicate *Igf2*$^{\mathrm{EpiKO}}$/C ratios.

Disease Models & Mechanisms • Supplementary information

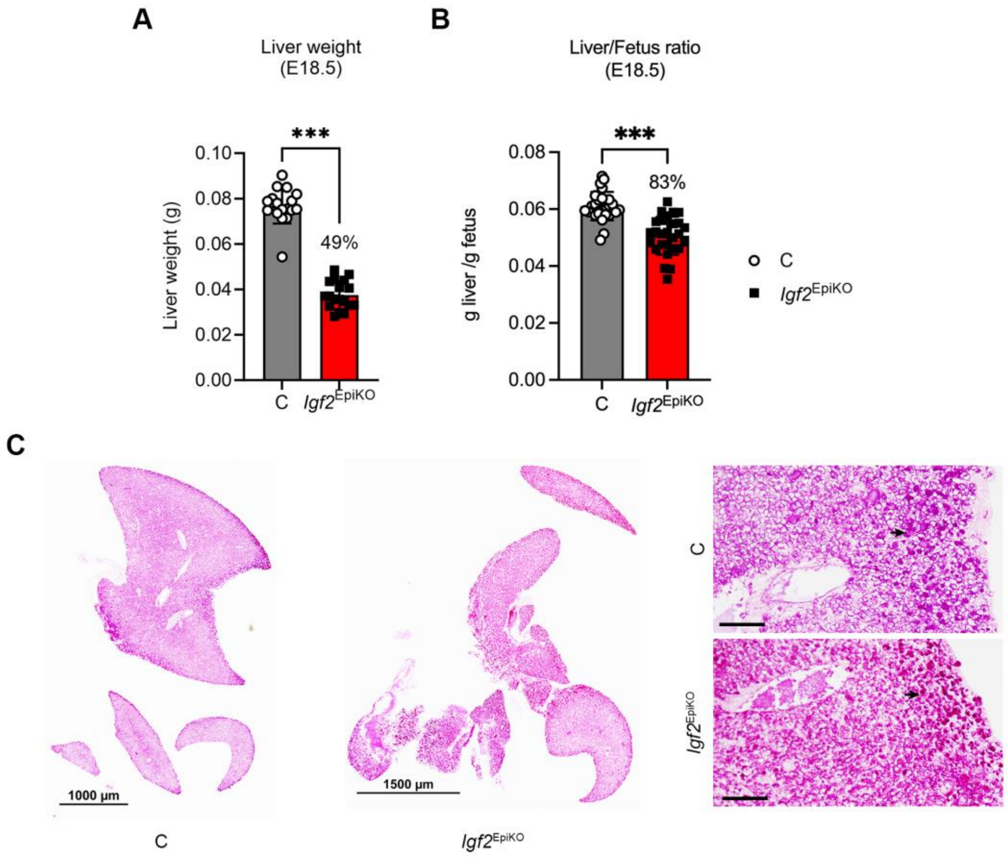


**Fig. S5. Liver weights and glycogen accumulation.** (A) Liver weights at E18.5 (n = 17 C and n = 18 *Igf2*$^{EpiKO}$ from N = 4 litters). (B) Proportion of livers as liver/fetus weight ratios at E18.5 (n = 27 C and n = 27 *Igf2*$^{EpiKO}$ from N = 7 litters). (C) Representative PAS stains for glycogen content in livers at E18.5 confirming similar patterns of glycogen deposits in the two genotypes. Images on the right side depict high-magnifications obtained in the images provided in the left side. For the low-magnification images, the sizes of each scale are indicated in the figures; for the high-magnification images, scale bars are 100 µm, arrows point to glycogen deposits (dark purple). For graphs (A) and (B), data is shown as individual values, with averages ± SD. *** - P < 0.001 by a Mann-Whitney test (A) or a student t test with Welch's correction (B); % values indicate *Igf2*$^{EpiKO}$/C ratios.

Disease Models & Mechanisms • Supplementary information

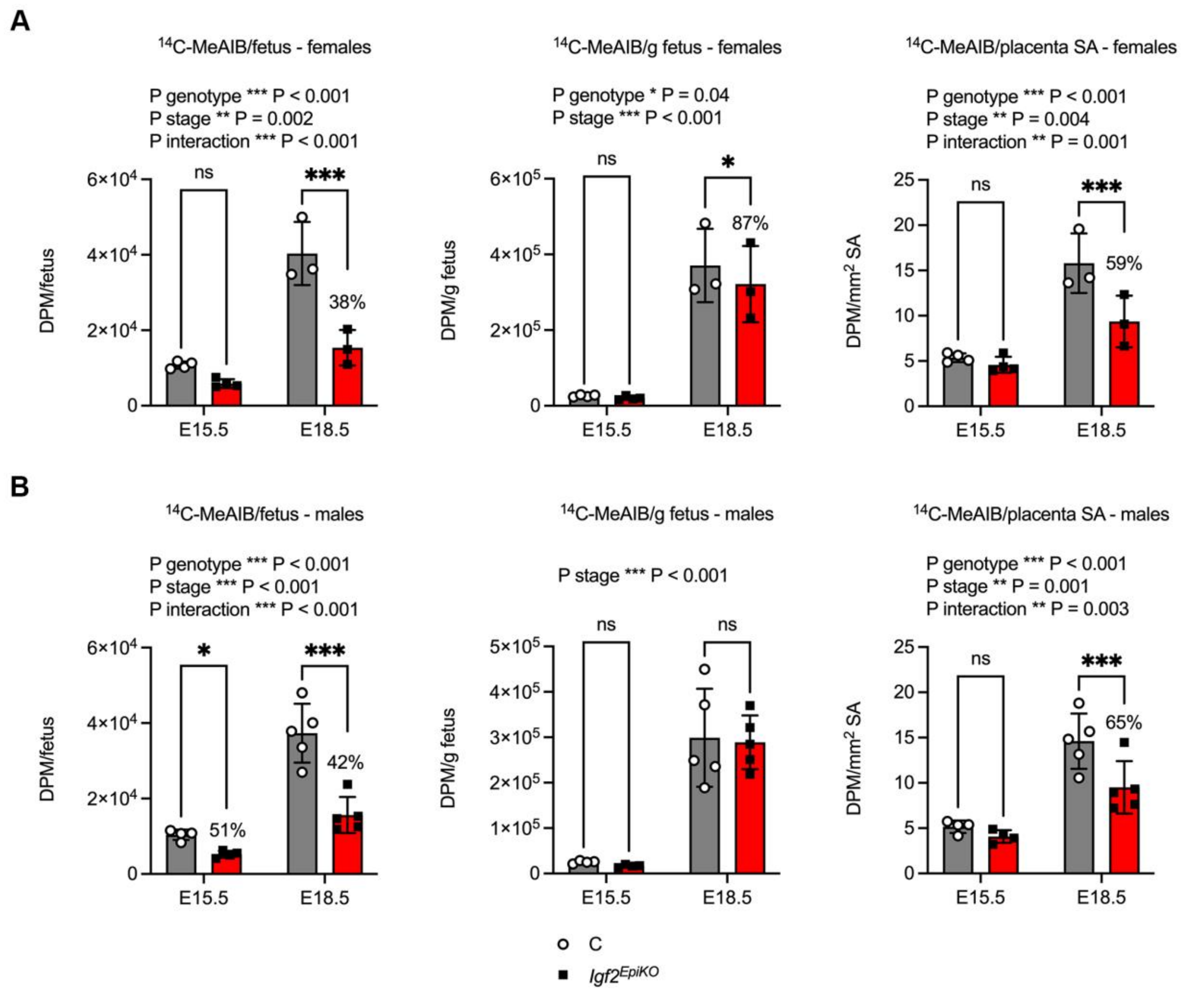


**Fig. S6. Analysis of placental transfer of $^{14}$C-methyl-aminoisobutyric acid ($^{14}$C-MeAIB) in relation to fetal sex.** (A) Measurements of disintegrations per minute (DPM) in the female fetuses, represented as counts per fetus (left panel), counts per gram fetal weight (middle panel) and counts per mm$^2$ labyrinthine zone surface area – SA (right panel). Data in (A) was collected in N = 4 litters at E15.5 (n = 9 C and n = 13 *Igf2*$^{\text{EpiKO}}$ mutants) and N = 3 litters at E18.5 (n = 6 C and n = 5 *Igf2*$^{\text{EpiKO}}$ mutants). (B) Measurements of disintegrations per minute (DPM) in the male fetuses, represented as counts per fetus (left panel), counts per gram fetal weight (middle panel) and counts per mm$^2$ labyrinthine zone surface area – SA (right panel). Data in (B) was collected in N = 4 litters at E15.5 (n = 11 C and n = 6 *Igf2*$^{\text{EpiKO}}$ mutants) and N = 5 litters at E18.5 (n = 11 C and n = 10 *Igf2*$^{\text{EpiKO}}$ mutants). For all graphs, data is presented as average values per litter, with mean values ± SD. P values shown above the graphs correspond to statistically significant repeated measures two-way ANOVA tests; ns – non-significant, * – P < 0.05, *** – P < 0.001 calculated by Sidak's multiple comparison tests following repeated measures two-way ANOVAs; % values indicate *Igf2*$^{\text{EpiKO}}$/C ratios.

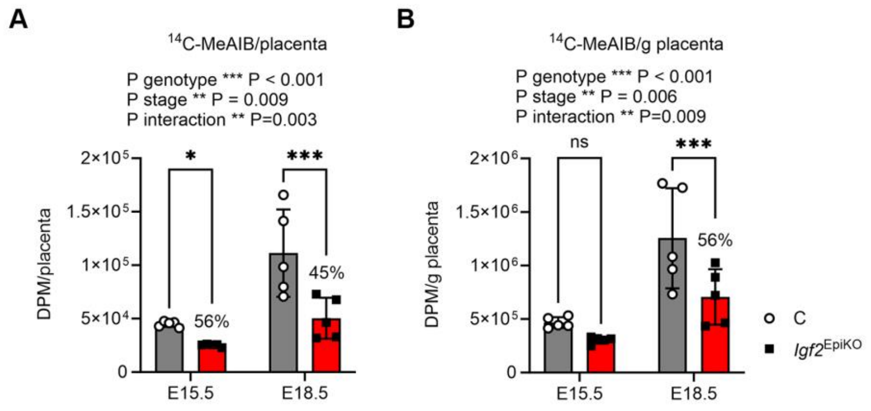


**Fig. S7. Placental accumulation of $^{14}$C-methyl-aminoisobutyric acid ($^{14}$C-MeAIB).** Measurements of DPM in placenta, represented as counts per placenta (A) or counts per gram placenta (B). For both graphs, data was collected in N = 5 litters at E15.5 (n = 22 C and n = 22 *Igf2*$^{EpiKO}$ mutants) and N = 5 litters at E18.5 (n = 22 C and n = 15 *Igf2*$^{EpiKO}$ mutants). Data are shown as average values per litter, with mean values ± SD. P values shown above the graphs correspond to statistically significant two-way ANOVA tests; ns – non-significant, * – P < 0.05, *** – P < 0.001 calculated by Sidak's multiple comparison tests following two-way ANOVAs; % values indicate *Igf2*$^{EpiKO}$/C ratios.

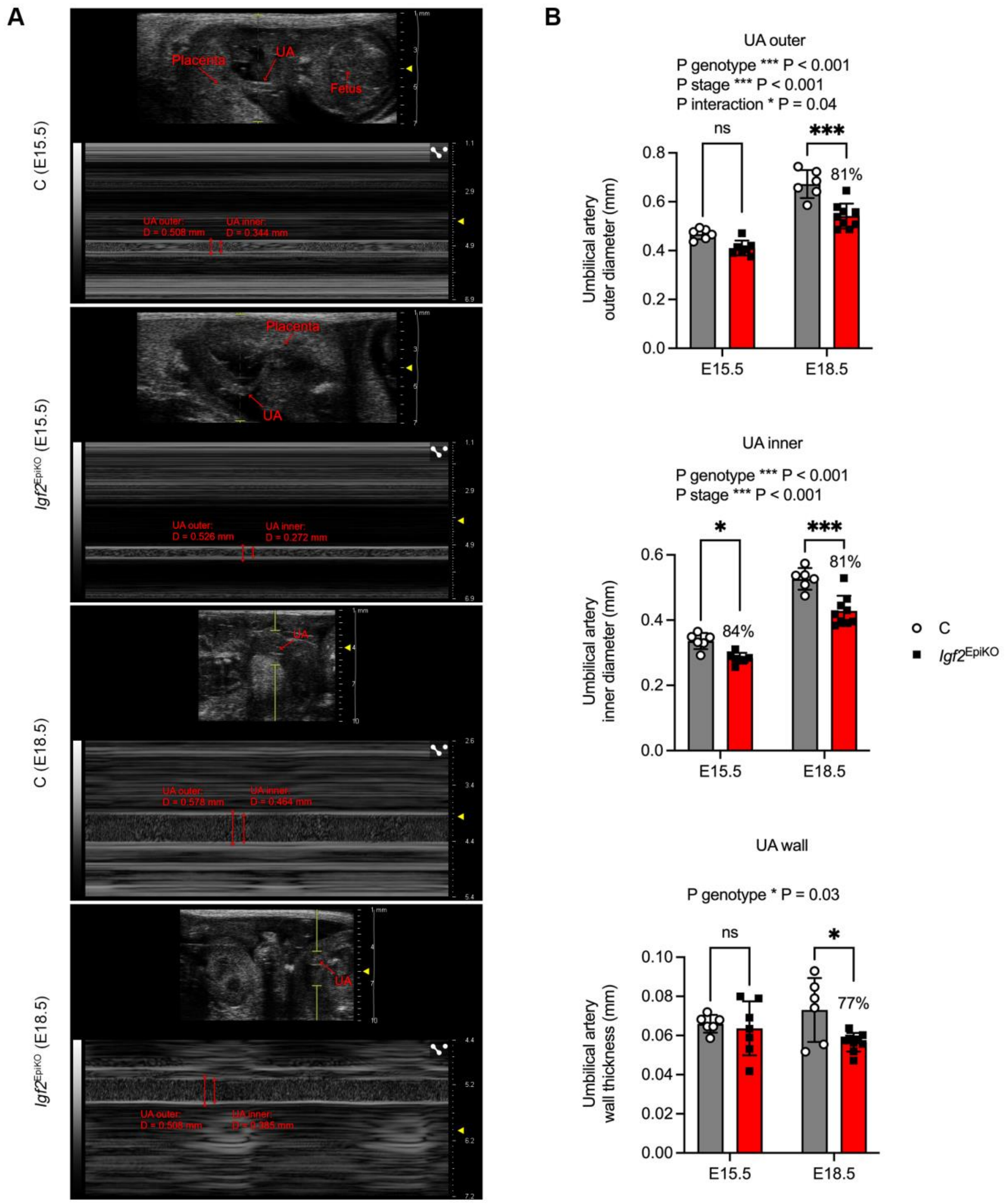


**Fig. S8. M-Mode ultrasonography analyses of umbilical artery in *Igf2*$^{\text{EpiKO}}$ mutants versus littermate controls.** (A) Representative M-Mode images depicting the umbilical artery of E15.5 and E18.5 C and *Igf2*$^{\text{EpiKO}}$ fetuses. (B) Measurements of umbilical artery (UA) outer (top) and inner (middle) diameters, as well as UA wall thickness (bottom). For all graphs, data was obtained in N = 3 litters at E15.5 (n = 6 C and n = 7 *Igf2*$^{\text{EpiKO}}$ mutants) and N = 3 litters at E18.5 (n = 6 C and n = 9 *Igf2*$^{\text{EpiKO}}$ mutants); data are shown as individual data points with mean values ± SD. P values shown above the graphs correspond

Disease Models & Mechanisms • Supplementary information

to statistically significant two-way ANOVA tests; ns – non-significant, * – $P < 0.05$, ** – $P < 0.01$, *** – $P < 0.001$ calculated by Sidak's multiple comparison tests following two-way ANOVAs; % values indicate *Igf2*$^{EpiKO}$/C ratios.

Disease Models & Mechanisms • Supplementary information

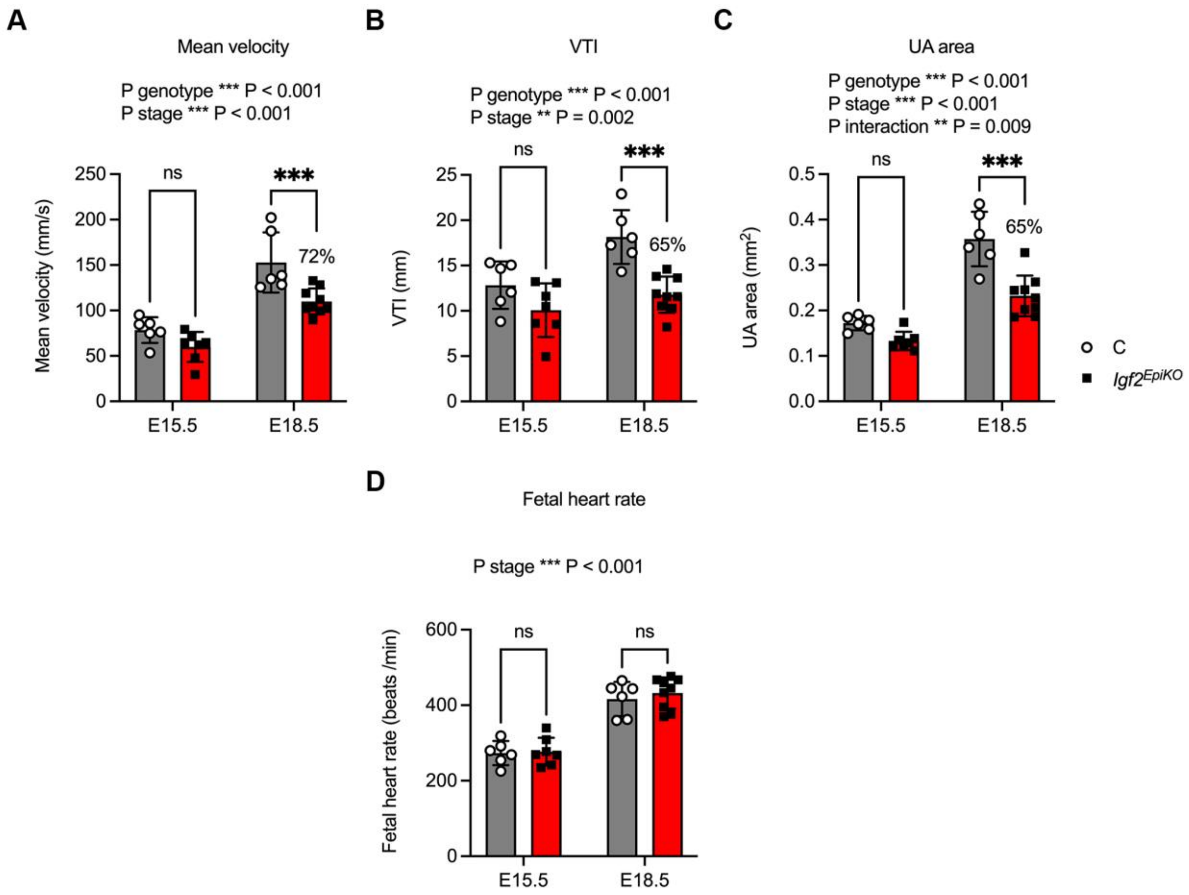


**Fig. S9. Additional ultrasonography measurements by PW Doppler.** Measurements of mean velocity (A), velocity-time integral (B), the area of the UA lumen (C) and fetal heart rate (D) at E15.5 and E18.5. Data was obtained in N = 3 litters at E15.5 (n = 6 C and n = 7 *Igf2*$^{EpiKO}$ mutants) and N = 3 litters at E18.5 (n = 6 C and n = 9 *Igf2*$^{EpiKO}$ mutants); data is shown as individual data points with mean values ± SD. The P values shown above the graphs corresponds to statistically significant two-way ANOVA tests; ns – non-significant; *** – P < 0.001 calculated by Sidak's multiple comparison tests following two-way ANOVAs; % values indicate *Igf2*$^{EpiKO}$/C ratios.

Disease Models & Mechanisms • Supplementary information

**Table S1. List of primers used for qRT-PCR**

| Gene | Primers sequences | Amplicon size (bp) |
|---|---|---|
| *Gapdh* | F: 5'-ACAACTCACTCAAGATTGTCAGCA-3'<br>R: 5'-ATGGCATGGACTGTGGTCAT-3' | 121 |
| *Pmm1* | F: 5'-ATCCGGGAGAAGTTTGTGGAA-3'<br>R: 5'-GCTGTCTTCATCCAGGCTGTC-3' | 144 |
| *Sdha* | F: 5'-TTCCGTGTGGGGAGTGTATTG-3'<br>R: 5'-ATTCTGCAGCTCCAGGGTCTC-3' | 135 |
| *Slc2a1* | F: 5'-AGTTCGGCTATAACACTGGTG-3'<br>R: 5'-GTGAGTGTGGTGGATGGGAT-3' | 112 |
| *Slc2a3* | F: 5'-GAGTCATCAATGCACCTGAGAC-3'<br>R: 5'-GAAGATGGCCACACATAAGGAC-3' | 125 |
| *Slc2a8* | F: 5'-GATGGTTGTCACTGGCATCC-3'<br>R: 5'-TGTTGAGTGAGGAGAAAACGTG-3' | 150 |
| *Slc38a1* | F: 5'-TTCACAGAAGTAGAAAACGGCC-3'<br>R: 5'-GTCGCATTTCCTTTTCTCCAGA-3' | 102 |
| *Slc38a2* | F: 5'-ATGCTGATCTTTATCCTCCCGT-3'<br>R: 5'-CATGCTTCCAATCATCACCACT-3' | 129 |
| *Slc38a4* | F: 5'-GGGACAGTATTCAGGACAGCTA-3'<br>R: 5'-CTTCTGACTTTCGGCATCTTCA-3' | 95 |
| *Slc1a5* | F: 5'-CATCATCCTGGAAGCAGTCAG-3'<br>R: 5'-GATCCACGTAACTCTGAAGCAG-3' | 143 |
| *Slc7a2* | F: 5'-TACACTGGTCTGGCAGAGTATC-3'<br>R: 5'-CACCATGACAAAGAGAAGGACC-3' | 150 |

Disease Models & Mechanisms • Supplementary information

**Table S2. Intra-observer and inter-observer coefficients of variation for all parameters measured by ultrasound imaging at E15.5 and E18.5**

| Parameter | Intra-observer CV (% ± 95% CI) | Inter-observer CV (% ± 95% CI) |
|---|---|---|
| E15.5 (n = 13 fetuses) | | |
| UA outer diameter | 1.25 ± 0.69 | 2.54 ± 1.62 |
| UA inner diameter | 2.74 ± 1.50 | 2.72 ± 1.15 |
| UA wall thickness | 6.94 ± 3.05 | 14.07 ± 5.23 |
| Fetal heart rate | 1.07 ± 0.36 | 1.64 ± 0.90 |
| PSV | 1.54 ± 0.73 | 2.44 ± 1.80 |
| EDV | 10.09 ± 4.12 | 12.04 ± 7.02 |
| Mean velocity | 2.77 ± 1.07 | 2.12 ± 1.38 |
| VTI | 1.07 ± 0.48 | N.A. |
| UA area | 5.49 ± 3.01 | 5.37 ± 2.26 |
| PI | 2.72 ± 1.01 | 1.78 ± 0.89 |
| RI | 0.59 ± 0.31 | 1.08 ± 0.48 |
| UA blood flow | 5.81 ± 2.69 | N.A. |
| E18.5 (n = 15 fetuses) | | |
| UA outer diameter | 1.08 ± 0.65 | 2.64 ± 1.07 |
| UA inner diameter | 1.10 ± 0.50 | 2.28 ± 0.95 |
| UA wall thickness | 6.04 ± 2.78 | 7.85 ± 2.98 |
| Fetal heart rate | 1.19 ± 0.80 | 0.61 ± 0.19 |
| PSV | 1.12 ± 0.47 | 0.94 ± 0.69 |
| EDV | 4.36 ± 2.28 | 4.02 ± 1.74 |
| Mean velocity | 2.17 ± 0.70 | 2.51 ± 0.75 |
| VTI | 1.43 ± 0.53 | N.A. |
| UA area | 2.23 ± 1.00 | 4.55 ± 1.89 |
| PI | 2.07 ± 0.77 | 2.51 ± 0.75 |
| RI | 0.52 ± 0.21 | 0.42 ± 0.13 |
| UA blood flow | 3.29 ± 1.29 | N.A. |

CV – coefficient of variation; CI – confidence interval; UA – umbilical artery; PSV – peak-systolic velocity; EDV – end-diastolic velocity; VTI – velocity time integral; PI – pulsatility index; RI – resistivity index

Disease Models & Mechanisms • Supplementary information
